# In Vivo and In Vitro Characterization of the Recently Emergent PRRSV 1-4-4 L1C Variant (L1C.5) in Comparison with Other PRRSV-2 Lineage 1 Isolates

**DOI:** 10.3390/v15112233

**Published:** 2023-11-09

**Authors:** Gaurav Rawal, Marcelo N. Almeida, Phillip C. Gauger, Jeffrey J. Zimmerman, Fangshu Ye, Christopher J. Rademacher, Betsy Armenta Leyva, Berenice Munguia-Ramirez, Grzegorz Tarasiuk, Loni L. Schumacher, Ethan K. Aljets, Joseph T. Thomas, Jin-Hui Zhu, Jolie B. Trexel, Jianqiang Zhang

**Affiliations:** 1 Department of Veterinary Diagnostic and Production Animal Medicine, College of Veterinary Medicine, Iowa State University, Ames, IA 50011, USA; grawal@iastate.edu (G.R.); malmeida@iastate.edu (M.N.A.); pcgauger@iastate.edu (P.C.G.); jjzimm@iastate.edu (J.J.Z.); cjrdvm@iastate.edu (C.J.R.); betsyarl@iastate.edu (B.A.L.); bmunguia@iastate.edu (B.M.-R.); tarasiuk@iastate.edu (G.T.); llschum@iastate.edu (L.L.S.); ealjets@iastate.edu (E.K.A.); jtthomas@iastate.edu (J.T.T.); miazjh@iastate.edu (J.-H.Z.); joliet@iastate.edu (J.B.T.); 2 Department of Statistics, Iowa State University, Ames, IA 50011, USA; fye@iastate.edu

**Keywords:** porcine reproductive and respiratory syndrome virus, PRRSV, 1-4-4 L1C variant, L1C.5, L1C.1, experimental study, virulence, transmissibility, brain infection

## Abstract

The recently emerged PRRSV 1-4-4 L1C variant (L1C.5) was in vivo and in vitro characterized in this study in comparison with three other contemporary 1-4-4 isolates (L1C.1, L1A, and L1H) and one 1-7-4 L1A isolate. Seventy-two 3-week-old PRRSV-naive pigs were divided into six groups with twelve pigs/group. Forty-eight pigs (eight/group) were for inoculation, and 24 pigs (four/group) served as contact pigs. Pigs in pen A of each room were inoculated with the corresponding virus or negative media. At two days post inoculation (DPI), contact pigs were added to pen B adjacent to pen A in each room. Pigs were necropsied at 10 and 28 DPI. Compared to other virus-inoculated groups, the L1C.5-inoculated pigs exhibited more severe anorexia and lethargy, higher mortality, a higher fraction of pigs with fever (>40 °C), higher average temperature at several DPIs, and higher viremia levels at 2 DPI. A higher percentage of the contact pigs in the L1C.5 group became viremic at two days post contact, implying the higher transmissibility of this virus strain. It was also found that some PRRSV isolates caused brain infection in inoculation pigs and/or contact pigs. The complete genome sequences and growth characteristics in ZMAC cells of five PRRSV-2 isolates were further compared. Collectively, this study confirms that the PRRSV 1-4-4 L1C variant (L1C.5) is highly virulent with potential higher transmissibility, but the genetic determinants of virulence remain to be elucidated.

## 1. Introduction

Porcine reproductive and respiratory syndrome virus (PRRSV) is the etiological agent of porcine reproductive and respiratory syndrome (PRRS), a disease significantly impacting the global swine industry. PRRSV is a single-stranded positive-sense RNA virus that is classified into two distinct species *Betaarterivirus suid 1* (virus name PRRSV-1) and *Betaarterivirus suid 2* (virus name PRRSV-2) in the genus *Betaarterivirus*, family *Arteriviridae*, order *Nidovirales* [1]. The PRRSV genome is approximately 15 kb in length and comprises 11 open reading frames (ORF), including ORF1a, ORF1b, ORF2a, ORF2b, ORF3, ORF4, ORF5a, ORF5, ORF6, ORF7, and a short transframe (TF) ORF in the nsp2 region [2]. The ORF1a and ORF1b encode replicase polyproteins that are processed into non-structural proteins (nsp1–nsp12), and TF ORF encodes proteins nsp2TF and nsp2N [2,3]. ORF2–ORF7 encode eight structural proteins GP2, E, GP3-GP5, ORF5a, M, and N.

PRRSV is distributed worldwide but with PRRSV-1 mainly present in Europe and PRRSV-2 mainly present in North America and Asia [4,5]. High genetic diversity has been observed within both PRRSV-1 and PRRSV-2 viruses [4]. In North America, restriction fragment length polymorphism (RFLP) typing based on cleavage patterns of three restriction enzymes (*Mlu*I, *Hinc*II, and *Sac*II) in ORF5 has been used to describe the genetic diversity of PRRSV-2 since the late 1990s [6] despite some shortcomings [7,8]. Phylogenetic classification systems based on ORF5 sequences have been developed and used to describe PRRSV-2 genetic diversity and relatedness [9,10,11]. Recently, based on an analysis of >82,000 global PRRSV-2 ORF5 sequences, Yim-im et al. [5] have further refined the phylogenetic classification system and proposed 11 lineages (L1–L11) and 21 sublineages (L1A–L1F and L1H–L1J, L5A and L5B, L8A–L8E, and L9A–L9E).

Since the first emergence of PRRSV in the late 1980s, global epidemics have been documented with emerging and re-emerging strains [12]. The periodical emergence of PRRSV-2 variants is not surprising due to its high mutation and recombination rate [12,13,14]. Some variants were detected transiently, whereas others established stable infections in swine populations. In the United States of America (USA), the first report of a virulent PRRSV-2 strain was in 1995 when PRRSV VR-2385 was isolated in Iowa with late-term abortion in sow farms and high mortality of piglets in farrowing and nursery units [15]. Other virulent PRRSV-2 strains emerged overtime, such as the MN184, SDSU73, JA142, 17198-6 [16], NADC30 [17], and 1-7-4 L1A variants, which emerged in the USA in 2013–2014 and were associated with high production losses [18,19]. In Asia, the notable example is the emergence of HP-PRRSV (representative isolates include JXA1, TJ, and HuN4; lineage 8) in China around 2006 with its subsequent spread to other Asian countries [20,21]. After that, in addition to HP-PRRSV, other PRRSV-2 variants, such as lineage 3 QYYZ-like, lineage 1 NADC30-like, lineage 1 NADC34-like, and numerous recombinant strains, have emerged in China and other Asian countries [21,22,23,24,25,26,27,28,29,30].

Beginning in October 2020, the high mortality and morbidity of pigs associated with PRRSV were observed in Iowa and Minnesota swine farms. ORF5 sequence analysis suggested that the PRRS viruses from these cases formed a distinct cluster within the sublineage L1C, and most of them had a 1-4-4 RFLP pattern; therefore, these PRRS viruses have been referred to as “PRRSV 1-4-4 L1C variant” [31,32]. Recently, the L1C viruses have been further classified into five groups (L1C.1–L1C.5) together with L1C-Others (1-4-4 L1C variant viruses correspond to L1C.5) [5]. Currently, the PRRSV 1-4-4 L1C variants have expanded to other swine-producing states like Missouri, Nebraska, Kansas, and South Dakota, causing piglet losses of around 8000 piglets per 1000 sows, which is more than double the losses caused by the PRRSV 1-7-4 L1A that emerged in 2013–2014 [33]. The perception was that this newly emerged PRRSV 1-4-4 L1C variant (L1C.5) is more virulent than other PRRSV strains based on field observations. However, no unequivocal experimental data were available to confirm that perception. Hence, the objectives of the current study were to (1) in vivo characterize the clinical impact, virulence, transmissibility, and antibody response of PRRSV 1-4-4 L1C.5 isolate in comparison with four other PRRSV isolates (1-4-4 L1C.1, 1-4-4 L1A, 1-4-4 L1H, and 1-7-4 L1A) in a weaned pig model under experimental conditions, and (2) in vitro characterize the growth characteristics of these isolates in ZMAC cells and compare their genomic sequences.

## 2. Materials and Methods

### 2.1. Cells

The ZMAC cell line was initially derived from the lung lavages of porcine fetuses [34], and we obtained the ZMAC cell line from the Aptimmune Biologics. ZMAC cells were cultured in the RPMI-1640 medium with L-glutamine and 25 mM HEPES (Corning, Oneonta, NY, USA) supplemented with 1× MEM non-essential amino acids (Corning), 4 mM sodium pyruvate (Corning), 2 mM L-glutamine (Corning), 0.81% glucose (Corning), 10% fetal bovine serum (Sigma-Aldrich, St. Louis, MO, USA), 0.01 µg/mL mouse macrophage colony-stimulating factor (mouse M-CSF, Shenandoah Biotechnology, Inc., Warwick, PA, USA), 0.05 mg/mL gentamicin, 100 unit/mL penicillin, 100 µg/mL streptomycin, and 0.25 µg/mL amphotericin. The MARC-145 cell line is a clone of the African monkey kidney cell line MA-104 [35]. MARC-145 cells were cultured in the regular RPMI-1640 medium supplemented with the final concentrations of 10% fetal bovine serum, 2 mM L-glutamine, 0.05 mg/mL gentamicin, 100 unit/mL penicillin, 100 µg/mL streptomycin, and 0.25 µg/mL amphotericin. MARC-145 and ZMAC cells were maintained at 37 °C incubator with 5% CO_2_.

### 2.2. Virus Isolates

Four RFLP 1-4-4 PRRSV-2 isolates selected for investigation in this study included L1C.5 (L1C variant) isolate USA/MN/01775GA/2021, L1C.1 isolate USA/NE/05828-3/2020, L1A isolate USA/85099/2018, and L1H isolate USA/81793-6/2019. One virulent PRRSV 1-7-4 L1A isolate, USA/IN/65239GA/2014, was included for comparison. These PRRSV isolates were selected based on the higher detection frequency of L1A, L1C, and L1H in recent years when comparing ORF5 sequences from 2006 to 2021 in the USA [5]. The five PRRSV-2 isolates used in this study were isolated and propagated in ZMAC cells and were titrated in both ZMAC and MARC-145 cell lines following the previously described protocol [36], and the results are summarized in Table 1. The five PRRSV isolates had PCR C_T_ values of 15.1–21.3, and infectious titers of 10^6^–10^6.75^ TCID_50_/mL in ZMAC cells and 10^3.5^–10^4.75^ TCID_50_/mL in MARC-145 cells. The ZMAC isolates at the appropriate dilutions were used for pig inoculation with 10^6^ TCID_50_ per pig, as described below.

**Figure 1:**
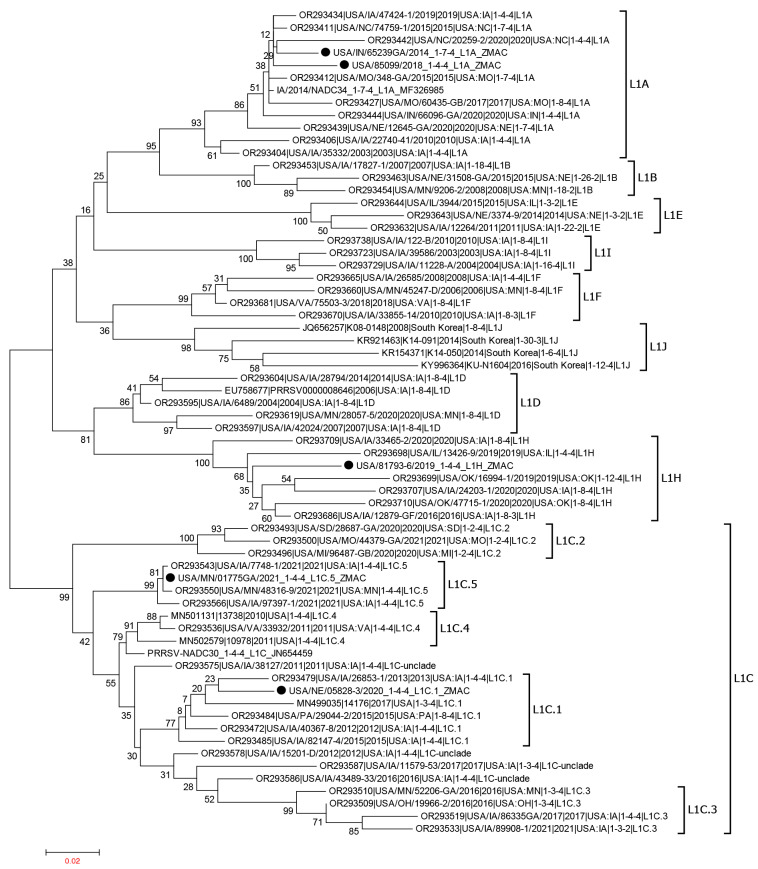
Phylogenetic tree based on ORF5 nucleotides of PRRSV-2 lineage 1 sequences. The representative L1A, L1B, L1C (LC.1, LC.2, L1C.3, L1C.4, L1C.5, and L1C-unclade), L1D, L1E, L1F, L1H, L1I, and L1J are depicted. The five PRRSV-2 isolates included in this study are shown using solid black bullet points.

The ORF5 sequences of these five PRRSV-2 isolates propagated in ZMAC cells were determined using the Sanger method, as previously described [37]. Their ORF5 sequences, together with the reference sequences representing different sublineages within lineage 1 [5], were aligned using MAFFT v7.407 [38], and the maximum likelihood tree was constructed using MEGA6 [39] with the bootstrap analysis of 1000 replicates. The locations of the five PRRSV isolates in the phylogenetic tree are shown in Figure 1.

### 2.3. Animal Source and Approval

Seventy-two PRRSV-naive pigs at three weeks of age were purchased, weaned, and transported to the Iowa State University Laboratory Animal Resources (ISU LAR) facility. The pigs were pre-screened eight days before delivery to confirm they were negative for PRRSV, porcine circovirus type 2 (PCV2), and porcine circovirus type 3 (PCV3) by PCR on serum samples, and negative for influenza A virus (IAV) and *Mycoplasma hyopneumoniae* (MHP) by PCR on nasal swabs. The pigs were also confirmed negative for PRRSV antibodies using a PRRS X3 ELISA kit (IDEXX Laboratories, Westbrook, ME) on serum samples. After pigs arrived at the ISU LAR facility, serum samples were tested again before inoculation to confirm they were still negative for PRRSV by PCR and PRRS X3 ELISA antibody assay. The pigs were also confirmed IAV negative in both nasal swabs and oral fluids using IAV PCR before inoculation. The study was approved by the Iowa State University Institutional Animal Care and Use Committee (approval number IACUC-21-124) and the Institutional Biosafety Committee (approval number IBC-21-054).

### 2.4. Animal Study Design

**Figure 2:**
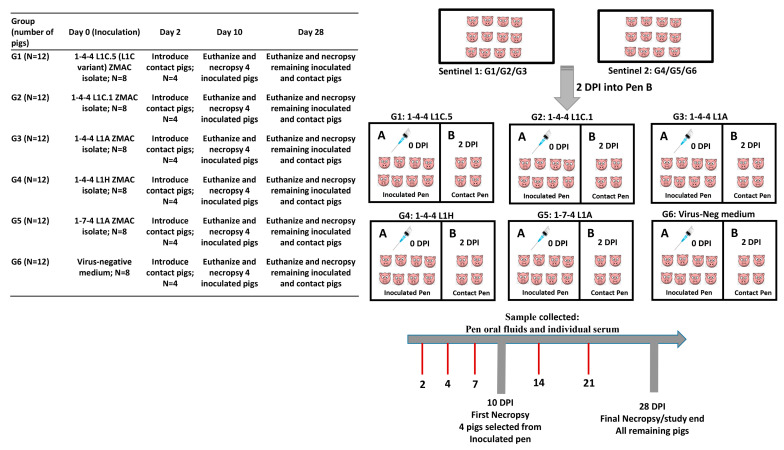
Experimental design of the pig study. On the (**left**), information of groups and the corresponding virus inoculum is provided. On the (**right**), a schematic diagram describes the pen in each room and the corresponding inoculation pigs and contact pigs.

The experimental design is shown in Figure 2. On the day of arrival, Excede^®^ (Ceftiofur crystalline free acid), at a dose of 5 mg/kg, was given to each pig intramuscularly in the post-auricular region of the neck. Each pig was microchipped to monitor body temperature. The microchips and microchip readers were purchased from Destron FearingTM. The microchip (Bio-ThermoTM) was intramuscularly implanted at the base of the left ear on the scutiform cartilage of each pig. All pigs were weighed and blocked by weight and then randomly divided into six groups with 12 pigs per group. Six rooms of the same size and condition were used in this study. Each room included two adjacent pens of equal measure (Pen A and Pen B). On the arrival day, forty-eight pigs were housed in Pen A of six rooms (8 pigs per group per room) as inoculation pigs, and the remaining 24 pigs were housed in two additional large rooms with 12 pigs per room to serve as contact pigs later on.

After seven days of acclimation, the inoculation pigs in groups 1 through 5 were inoculated with the respective ZMAC isolate at the appropriate dilution via the intramuscular route (2 mL/pig) in the neck muscle area and the intranasal route (2 mL/nostril) using mucosal atomization devices (MADs) fitted to a syringe that generated mist or sprays by manual pressure, which resulted in particle sizes of 30–100 μm [40], with each pig receiving 10^6^ TCID_50_ of the respective virus inoculum in total. The inoculation pigs in group 6 were inoculated with a virus-negative medium following the same administration routes and the same volume. At 2 days post inoculation (DPI; corresponding to 0 day post contact (DPC)), four contact pigs were added to Pen B in each of the six rooms (Figure 2). Four inoculated pigs per group were randomly selected and necropsied at 10 DPI to check for gross and microscopic lesions. If any pigs died or were euthanized due to severe body conditions before the scheduled days, pigs were necropsied accordingly. At 28 DPI, all the remaining pigs were euthanized and necropsied.

Daily temperature and clinical signs (coughing, anorexia and lethargy, and respiratory distress) were recorded. Each pig was recorded for clinical signs using scores from 0 to 3. For coughing, 0 = normal; 1 = mild coughing, occasional; 2 = moderate coughing, often; 3 = repetitive, productive cough. For anorexia and lethargy, 0 = normal; 1 = moderate activity, normal abdominal fill, mild interest in feed; 2 = ambulatory but slow movement, stands around, inactive, mildly gaunt and tucked flank; 3 = inactive and laying down, gaunt, flank is tucked up/flat, no interest in feed. For respiratory distress, 0 = normal; 1 = mild but increased respiration when active; 2 = moderate increased respiration at rest, mild dyspnea (labored breathing) when active; 3 = severe, constant, dyspnea, abdominal breathing, open-mouth breathing.

Pigs were weighed at -1 DPI, 10 DPI, 28 DPI, and any day when pigs had to be euthanized before the scheduled necropsy days. The weight difference between two time points were divided by the number of days in the interval to calculate the average daily weight gain (ADG). Serum and oral fluid samples were collected at 0, 2, 4, 7, 10, 14, 21, and 28 DPI. Blood was collected using BD Vacutainer™ SST™ tubes (Becton, Dickinson and Company, Franklin Lakes, NJ, USA) via the anterior vena cava and centrifuged at 3000 rpm for 15 min. The serum was aliquoted and stored at −80 °C until testing. Cotton ropes were used to collect oral fluids from each pen as previously described [41]. At necropsy, gross lung lesions were scored; fresh and formalin-fixed tissues were collected and included lung, tonsil, brain, tracheobronchial lymph nodes, thymus, heart, spleen, and kidney.

### 2.5. Sample Processing

Fresh lung, tonsil, and brain tissues were processed by placing 2.5 g of tissue into 22.5 mL of Dulbecco’s Modified Eagle’s Medium (DMEM, Sigma, Cibolo, TX, USA) in a 50 mL conical tube, followed by grinding for 30 s using a geno grinder homogenizer (Thermo Fisher Scientific, Waltham, MA, USA) to obtain a 10–20% solution. After centrifugation at 4200× *g* for 10 min, the tissue homogenates were harvested and stored at −80 °C until testing.

### 2.6. Nucleic Acid Extraction and Quantitative PRRSV Real-Time RT-PCR

Nucleic acids were extracted from 100 µL of clinical samples using a MagMAX Pathogen RNA/DNA extraction kit (Thermo Fisher Scientific, Waltham, MA, USA) and a Kingfisher Flex instrument (Thermo Fisher Scientific) following the manufacturer’s instructions. Nucleic acids were eluted into 90 µL of elution buffer. A commercial PRRSV RT-qPCR (VetMAX PRRSV NA&EU One-Step RT-PCR assay, Thermo Fisher Scientific), including the TaqMan Fast 1-Step RT-PCR master mix and primers and probes mix v2, was used following the previously described protocols [42]. A Ct < 37 was considered positive, and Ct ≥ 37 was considered negative for PRRSV. Standard curves generated using known concentrations of in vitro transcribed viral RNA provided in the assay kits were used to quantify the genomic copies of the virus in the samples.

### 2.7. PRRSV Antibody Measurement

A PRRS X3 ELISA antibody kit (IDEXX Laboratories, Westbrook, ME, USA) was used to test serum samples collected at 0, 7, 14, 21, and 28 DPI from four virus-inoculated pigs that survived after 10 DPI and all contact pigs. According to the manufacturer, samples with sample-to-positive (S/P) ratios ≥0.4 were considered positive for antibody against PRRSV.

The same set of serum samples was tested by indirect fluorescent antibody (IFA) assay [43] using the ISU-P isolate [44] as the indicator virus.

The same set of serum samples was also tested for neutralizing antibodies using the fluorescent focus neutralization (FFN) assay and the homologous virus isolate as the indicator virus. For example, serum samples from the L1C.5 (L1C variant) group were tested for neutralizing antibody against L1C.5 isolate MN/01775GA/2021. Serum samples were first inactivated at 56 °C for 30 min, then 2-fold serially diluted from 1:2 to 1:256 dilutions in 96-well plates with a volume of 100 µL per well after dilution. Subsequently, 100 µL of the respective PRRSV isolate was mixed with an equal volume of diluted sera (in the serum–virus mixture, the serum dilution was adjusted from 1:4 to 1:512). After incubation for 1 h at 37 °C with 5% CO_2_, 100 µL of the serum–virus mixture (containing 100–200 TCID_50_ of virus) was transferred to 96-well plates with a MARC-145 cell monolayer. The plates were incubated at 37 °C with 5% CO_2_ for 48 h, fixed with cold 80% acetone, and stained with the mixture of PRRSV N protein-specific monoclonal antibody SDOW17 and SR30-conjugated to FITC (Rural Technologies Inc., Brookings, SD, USA) at a 1:100 dilution for 60 min. The staining was examined under a fluorescent microscope. The reciprocal of the highest serum dilution resulting in >90% reduction in staining compared to the antibody-negative serum control was defined as the FFN antibody titer of the serum sample. An FFN antibody titer of ≥8 was considered positive.

### 2.8. Bacterial Culture on Lung and Brain Homogenates

In order to determine if pigs were infected with any bacteria, the lung and brain homogenate samples from all 72 pigs in the study were submitted for general bacterial culture at the Bacteriology Section of Iowa State University Veterinary Diagnostic Laboratory (ISU VDL).

### 2.9. Gross Lung Pathology, Histopathology, and Immunohistochemistry

At necropsy, lung tissue from each pig was examined in a blind fashion by a single pathologist and given a subjective score for the severity of gross lung lesions using an established scoring system [15] that estimated the percentage of lung tissues affected by pneumonia and consolidation. Formalin-fixed tissues of the lung (two sections from the caudal lobe and one from each of middle, accessory, and cranial lung lobes), tonsils, tracheobronchial lymph nodes, thymus, heart, spleen, kidney, and brain were submitted to ISU VDL for histopathological examination. Lung and brain tissues were also tested by PRRSV immunohistochemistry (IHC). For microscopic and IHC lung scores, one pathologist blind to treatments scored each of the five lung sections for all pigs and then averaged those numbers to give a unique value per pig. The microscopic sections were examined and assigned a score for the severity of interstitial pneumonia (0 to 6) as previously described [15,45]. The IHC score was based on a 0 to 3 system, as previously described [46].

### 2.10. Whole Genome Sequence Analysis of PRRSV Isolates

The whole genome sequences of the PRRSV-2 isolates MN/01775GA/2021 (1-4-4 L1C.5), NE/05828-3/2020 (1-4-4 L1C.1), 85099/2018 (1-4-4 L1A), 81793/2019 (1-4-4 L1H), and IN/65239//2014 (1-7-4 L1A) propagated in ZMAC cells were determined via next-generation sequencing (NGS) technology following the previously described procedures [47]. The whole genome sequences were deposited into GenBank with the accession numbers OR634972 (USA/MN/01775GA/2021), OR634973 (USA/NE/05828-3/2020), OR634974 (USA/85099/2018), OR634975 (USA/81793-6/2019), and OR634976 (USA/IN/65239GA/2014). The sequence identities of these isolates were determined at the whole genome level and at the individual gene or protein level using the MAFFT alignment of the MegAlign Pro 17 program in DNASTAR Lasergene 17 software. The N-glycosylation sites on GP2, GP3, GP4, and GP5 proteins were predicted using netNGlyc-1.0 software [48].

### 2.11. Multistep Growth Curve in Cell Culture

For in vitro characterization, the growth curves of five PRRSV-2 lineage 1 isolates included in this study were compared in ZMAC cell line derived from primary alveolar macrophages. Each stock virus was diluted to 1 × 10^6^ TCID_50_/mL based on the titration conducted in ZMAC cells. Monolayers of ZMAC cells grown in 24-well plates were inoculated with each PRRSV-2 isolate at a multiplicity of infection (moi) of 0.1. After 1 h absorption at 37 °C in 5% CO_2_ incubator, the virus inoculum was discarded, and two milliliters of fresh medium was added to each well of cells, and this time point was designated time zero with respect to infection. Seven 24-well plates were used for the entire experiments, with one plate for each time point and duplicate wells for each virus at each time point. The cell plates were incubated at 37 °C with 5% CO_2_. At 0, 6, 12, 24, 48, 72, and 96 h post infection (hpi), the plates were frozen at −80 °C. After one freeze–thaw cycle, the cell lysates were centrifuged at 1200× *g* for 10 min, and the supernatant was saved at −80 °C for titration. Each isolate at each hpi was titrated using both aliquots. The supernatants were 10-fold serially diluted and titrated in ZMAC cells grown in 96-well plates with triplicate wells per dilution. Virus titers were determined according to the Reed and Muench method [49] and expressed as TCID_50_/mL.

### 2.12. Statistical Analysis

Two-way ANOVA with interaction was applied for analyzing virus titers in a multi-step growth curve. The two factors in the analysis were virus groups and hours post infection. Temperatures, anorexia and lethargy scores, virus loads in serum determined by quantitative PRRSV real-time RT-PCR. PRRSV antibody (PRRS X3 ELISA) S/P ratios, PRRSV IFA antibody titers converted into log2 format, and PRRSV FFN antibody titers converted into log2 format over time were analyzed using a linear mixed model. The ADG, PRRSV RNA levels in tissues (e.g., lung, tonsil, and brain), and gross lung lesion scores were analyzed using one-way ANOVA with Tukey–Kramer HSD. The microscopic lesion scores in lungs and IHC scores in lung and brain tissues were analyzed using a linear mixed model. No statistical analysis was carried out on oral fluid data. For all analyses, SAS was used, and a *p*-value < 0.05 was considered significant.

## 3. Results

### 3.1. Clinical Observations (Anorexia and Lethargy, Mortality, Microchip Temperature, and ADG) in Inoculated Pigs

The mock-inoculated pigs did not show clinical signs of illness throughout the experiment. Temperature and respiratory disease scores in this negative control group were within the normal ranges.

**Figure 3:**
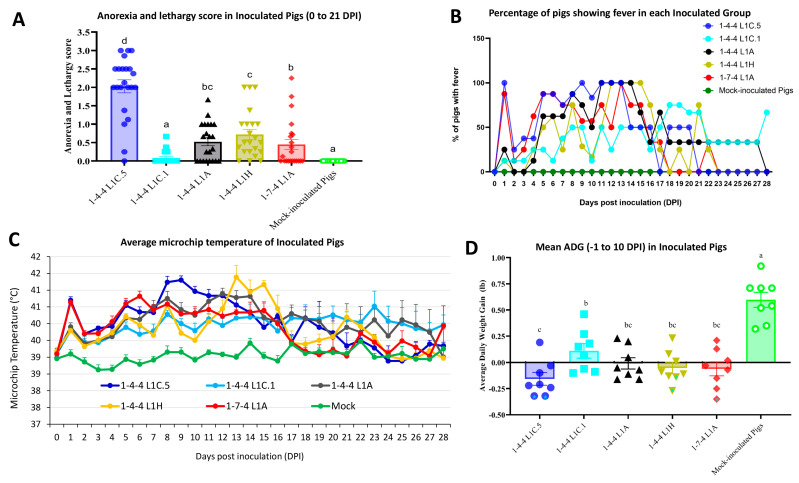
Clinical observations in inoculated pigs. (**A**) The overall average anorexia and lethargy scores in different groups from 0 to 21 DPI. For each group, the available pigs were averaged for their anorexia and lethargy scores for each day. Therefore, there are 22 spots for each group corresponding to each day from 0 to 21 DPI. (**B**) Percentage of pigs showing fever (>40 °C) in each group at each time point. (**C**) Microchip body-temperature changes of inoculated pigs over time. The mean microchip temperature in degree Celsius is shown on the *Y*-axis. (**D**) Mean average daily weight gain (ADG) of inoculated pigs between −1 DPI and 10 DPI, with significance denoted by letters. Pigs that died or were euthanized due to severe body conditions before 10 DPI are shown by * in (**D**). Labels with different letters indicate significant differences; for example, a and b have a significant difference, but a and ab have no significant difference.

Pigs inoculated with the 1-4-4 L1C.5 isolate or 1-7-4 L1A isolate became more lethargic and were off-feed faster than other virus-inoculated groups. Regarding the anorexia and lethargy scores, the 1-4-4 L1C.5 isolate-inoculated group was significantly different from other virus-inoculated groups at different time points, such as 1, 4, 5, 8–13, and 16–21 DPI. The overall anorexia and lethargy scores in different inoculation groups are shown in Figure 3A. Based on an overall statistical analysis of the anorexia and lethargy scores from 0 to 21 DPI, pigs inoculated with the 1-4-4 L1C.5 isolate had significantly higher scores (a mean score of 2) compared to other groups; the 1-4-4 L1C.1 inoculation group had the lowest scores among all virus-inoculated groups.

The mortality was calculated by counting the pigs that naturally died or were euthanized due to severe body conditions based on the IACUC protocol. As shown in Table 2, pigs inoculated with the 1-4-4 L1C.5 isolate had higher mortality (6 out of 8 pigs) than other virus-inoculated groups (1–2 pigs out of 8 pigs).

The 1-4-4 L1C.5 virus-inoculated group had a higher percentage of pigs with fever (>40 °C) during 0–10 DPI (Figure 3B). At 1 DPI, 100% and 90% of inoculated pigs in the 1-4-4 L1C.5 group and 1-7-4 L1A group, respectively, developed fever, which is higher than other groups. Subsequently, the number of pigs with fever in these two groups (1-4-4 L1C.5 and 1-7-4 L1A) decreased during 2–3 DPI and increased again starting from 4 or 5 DPI, with 50–100% of pigs having fever again during 5–16 DPI (Figure 3B). A higher number of pigs had fever in the 1-4-4 L1C.5 inoculation group than in the 1-7-4 L1A inoculation group during 9–12 DPI, but it was opposite during 14–15 DPI (Figure 3B). For groups inoculated with the 1-4-4 L1C.1, 1-4-4 L1A, or 1-4-4 L1H isolate, very few pigs developed fever during 0–4 DPI; the number of pigs developing fever started to increase at 5 DPI for 1-4-4 L1A and 1-4-4 L1H inoculation groups and at 7 DPI for 1-4-4 L1C.1 inoculation group (Figure 3B). A higher number of pigs had fever in the 1-4-4 L1C.1 inoculation group during 18–20 DPI, which was different from other inoculation groups (Figure 3B). Consistently, 1-4-4 L1C.5 and 1-7-4 L1A inoculation groups had significantly higher average temperatures than other virus-inoculated groups at 1 DPI (Figure 3C). The average temperatures in 1-4-4 L1C.5 inoculation group were similar to those in 1-7-4 L1A inoculation group during 2–7 DPI but were significantly higher than the latter during 8–10 DPI (Figure 3C). The 1-4-4 L1H inoculation group had a delayed temperature peak (13–15 DPI) compared to other inoculation groups. Overall, the 1-4-4 L1C.1 inoculation group had low average temperature throughout the study (Figure 3C). All virus-inoculated groups had significantly higher average temperatures than the mock-inoculated group at the time points 1 and 3–16 DPI.

All virus-inoculated groups had significantly lower ADG than the mock-inoculation group between −1 and 10 DPI (Figure 3D). The mean ADG in 1-4-4 L1C.5 inoculation group was significantly lower than 1-4-4 L1C.1 inoculation group but not statistically different from the other three virus inoculation groups between −1 and 10 DPI (Figure 3D).

### 3.2. Viral Loads in Serum and Oral Fluid Samples of Inoculated Pigs

**Figure 4:**
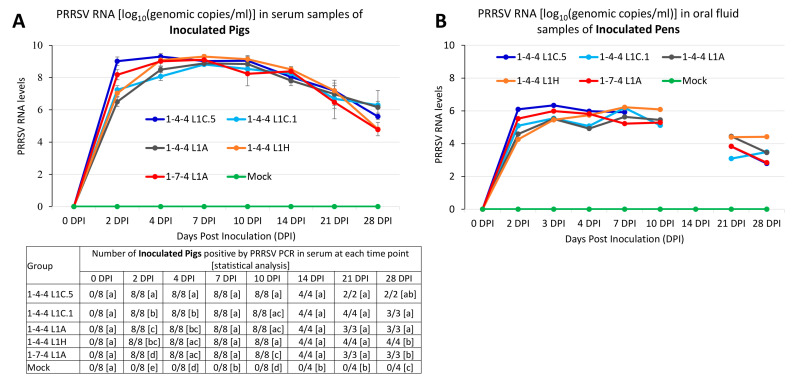
PRRSV RNA load in different specimens of inoculated pigs. (**A**) PRRSV RNA detected in serum samples of inoculated pigs by quantitative real-time RT-PCR. The average PRRSV RNA levels in the unit of log_10_(genomic copies/mL) of each group in serum samples at each time point are shown. The number of pigs confirmed positive by PRRSV PCR and statistical analysis are shown in the bottom. Pigs that died or euthanized between 8 and 10 DPI were included for counting at 10 DPI. Similarly, dead pigs between 11 and 14 DPI were counted at 14 DPI, dead pigs between 15 and 21 DPI were counted at 21 DPI, and dead pigs between 21 and 28 DPI were counted at 28 DPI. Labels with different letters indicate significant differences; for example, a and b have a significant difference, but a and ab have no significant difference. (**B**) PRRSV RNA log_10_(genomic copies/mL) in pen-based oral fluids of inoculated pigs. Oral fluids could not be collected at some time points due to the inactivity of pigs.

Pigs in all groups were negative by PRRSV quantitative real-time RT-PCR at 0 DPI in serum samples. After 2 DPI, serum samples from all eight inoculated pigs in each virus-inoculated group became PRRSV PCR-positive (Figure 4A). At 2 DPI, the 1-4-4 L1C.5 virus-inoculated group had a significantly higher viremia level than all other virus-inoculated groups, and the 1-7-4 L1A inoculation group had a significantly higher viremia level than the 1-4-4 L1C.1, 1-4-4 L1A, and 1-4-4 L1H inoculation groups (Figure 4A). During 4–28 DPI, there were some significant differences in viremia levels between groups, but no consistent trends were observed. For example, the 1-4-4 L1C.5 virus-inoculated group had a significantly higher viremia level than 1-4-4 L1C.1 and 1-4-4 L1A at 4 DPI. The 1-4-4 L1C.5 and 1-4-4 L1H virus-inoculated groups had significantly higher viremia levels compared to the 1-7-4 L1A at 10 DPI. At 28 DPI, the 1-4-4 L1C.1 and 1-4-4 L1A virus-inoculated groups had significantly higher viremia levels than 1-4-4 L1H and 1-7-4 L1A. No statistical difference in viremia level was found among virus-inoculated groups at 7, 14, and 21 DPI. All virus-inoculated groups were significantly different compared to the mock-inoculated group at 2, 4, 7, 10, 14, 21, and 28 DPI.

Similar to the viremia level, the 1-4-4 L1C.5 virus-inoculated pen had a numerically higher virus load in oral fluid than all other virus-inoculated pens, while the 1-7-4 L1A virus-inoculated pen had a numerically higher virus load than 1-4-4 L1C.1, 1-4-4 L1A, and 1-4-4 L1H virus-inoculated pens at 2 and 3 DPI (Figure 4B). The 1-4-4 L1H virus-inoculated pen had a higher virus load than all other virus-inoculated pens at 7 and 28 DPI. Notably, no statistical analysis was conducted on viral loads in oral fluid samples because there was only one oral fluid sample from each inoculation pen at each time point. Also, no oral fluid was collected from the 1-4-4 L1C.5 virus-inoculated pen at 10 DPI. At 14 DPI, no oral fluids were collected from any virus-inoculated pen.

### 3.3. Gross Lung Lesions of Inoculated Pigs

**Figure 5:**
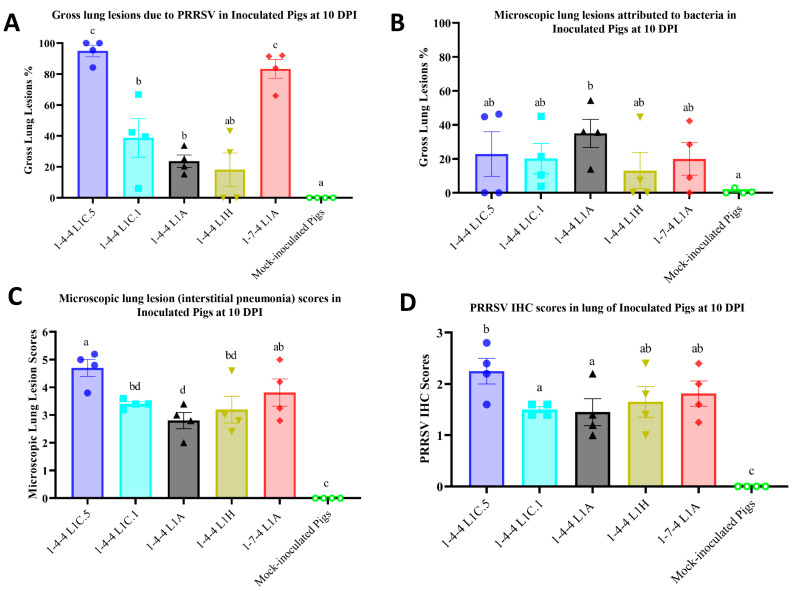
Gross lesions, microscopic lesions, and PRRSV immunohistochemistry (IHC) scores in lung tissues of inoculated pigs at 10 DPI. Percentage of macroscopic lung lesions due to PRRSV in inoculated pigs (**A**) and percentage of macroscopic lung lesions due to bacteria in inoculated pigs (**B**) are shown. (**C**) Microscopic lung lesion scores (in the range of 0–6) in inoculated pigs at 10 DPI. (**D**) PRRSV IHC staining scores (in the range of 0–3) in lung tissues of inoculated pigs at 10 DPI. A cluster graph was used to present the data with standard error of the mean. The statistical analysis was conducted between groups, with significance denoted by letters on the individual plot. Labels with different letters indicate significant differences; for example, a and b have a significant difference, but a and ab have no significant difference.

**Figure 6:**
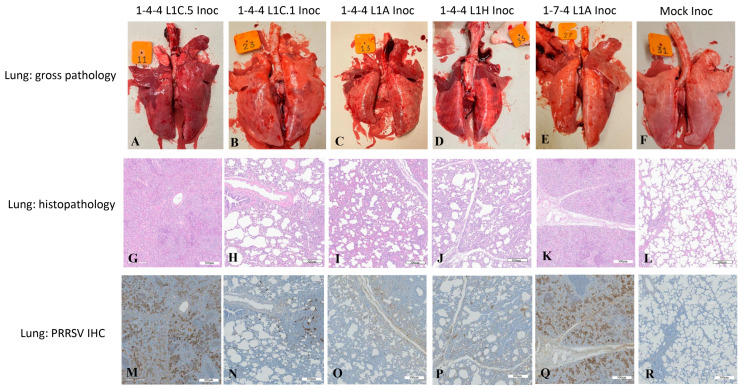
Representative images showing gross lesions, microscopic lesions, and PRRSV immunohistochemistry (IHC) staining in lung tissues at 10 days post inoculation (DPI). The inoculation groups are shown at the top. Gross lung pathology, microscopic lung lesions, and PRRSV IHC staining in lung tissues are exemplified in (**A**–**F**), (**G**–**L**), and (**M**–**R**), respectively.

When compared to 1-4-4 L1C.1, 1-4-4 L1A, and 1-4-4 L1H virus-inoculated groups, pigs in the 1-4-4 L1C.5 and 1-7-4 L1A inoculation groups had significantly higher gross lung lesions attributed to PRRSV infection at 10 DPI (Figure 5A). But, there were no significant differences regarding gross lung lesions attributed to PRRSV infection among 1-4-4 L1C.1, 1-4-4 L1A, and 1-4-4 L1H inoculation groups, although the gross lung lesions were significantly higher in 1-4-4 L1C.1 and 1-4-4 L1A inoculation groups compared to the mock-inoculation group (Figure 5A). Representative images of gross lung lesions are shown in Figure 6A–F. Gross lung lesions, i.e., lung consolidation attributed to bacterial infection, were also assessed in all inoculation groups at 10 DPI, but overall, there were no significant differences between them (Figure 5B). Gross lung lesions were minimal at 28 DPI in all inoculation groups.

Furthermore, lung homogenate samples from all inoculated pigs were subject to bacterial culture. For most pigs, there was no significant bacteria growth, although a low–moderate–high number of colonies for *Streptococcus suis* and *Trueperella pyogenes* was detected in a few pigs across all inoculation groups (one pig in mock, one pig in 1-4-4 L1H, two pigs in 1-4-4 L1A, one pig in 1-4-4 L1C.1, two pigs in 1-4-4 L1C.5, and two pigs in 1-7-4 L1A inoculation groups).

### 3.4. Lung Microscopic Lesions and Immunohistochemistry Scores in Inoculated Pigs

A significantly higher mean microscopic lung lesion score was observed in the 1-4-4 L1C.5 inoculation group compared to the 1-4-4 L1C.1, 1-4-4 L1A, and 1-4-4 L1H inoculation groups, but there was no significant difference regarding the microscopic lung lesions between the 1-4-4 L1C.5 and 1-7-4 L1A inoculation groups at 10 DPI (Figure 5C). All virus inoculation groups had significantly higher microscopic lung lesions than the mock-inoculated group. Similarly, the mean PRRSV IHC lung score in L1C.5 inoculation group was significantly higher than the 1-4-4 L1C.1 and 1-4-4 L1A inoculation groups but was insignificantly different from the 1-4-4 L1H and 1-7-4 L1A inoculation groups at 10 DPI (Figure 5D). All virus-inoculated groups had significantly higher lung PRRSV IHC scores compared to the mock-inoculated group at 10 DPI (Figure 5D). Representative images of microscopic lung lesions and PRRSV IHC staining in lung tissues are shown in Figure 6G–R. Microscopic lung lesions and PRRSV IHC staining in lung tissues at 28 DPI were not remarkable in all inoculation groups (Supplemental Figure S1).

### 3.5. PRRSV RNA Load in Tissue Samples of Inoculated Pigs

**Figure 7:**
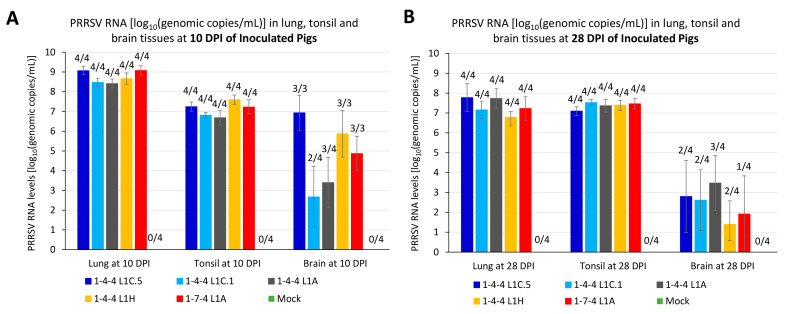
PRRSV RNA load in different specimens of inoculated pigs. (**A**) PRRSV RNA log_10_(genomic copies/mL) in lung, tonsil, and brain tissues of inoculated pigs necropsied at 10 DPI or during 9–10 DPI. (**B**) PRRSV RNA log_10_(genomic copies/mL) in lung, tonsil, and brain tissues of inoculated pigs necropsied at 28 DPI or during 10–28 DPI. Number of pigs confirmed positive by PRRSV PCR is indicated on top of each histogram.

A PRRSV quantitative real-time RT-PCR was used to detect PRRSV RNA in lung, tonsil, and brain tissue samples in inoculated pigs necropsied at 10 DPI or during 9–10 DPI (Figure 7A) and at 28 DPI or during 10–28 DPI (Figure 7B). All lung and tonsil samples from each virus inoculation group were PRRSV PCR-positive at both time points. Brain tissues were not collected from the three pigs that died naturally before 10 DPI, all of which were from the 1-4-4 L1C.5, 1-4-4 L1H, and 1-7-4 L1A inoculation groups; hence, only three brain samples from each of those groups were available at 10 DPI. For the brain samples, 2/4 up to 3/3 pigs at 10 DPI and 1/4 up to 3/4 pigs at 28 DPI were PRRSV PCR-positive among all virus-inoculated groups (Figure 7A,B).

Although numerical differences were observed among some groups, PRRSV RNA loads in lung homogenates were not significantly different among five virus-inoculated groups at 10 DPI or 28 DPI. Similar results and conclusions were obtained for tonsil and brain homogenates.

### 3.6. PRRSV Immunohistochemistry Staining in Brain Tissues

**Figure 8:**
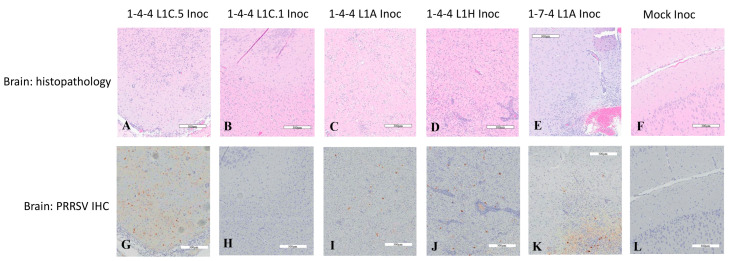
Representative images showing microscopic lesions and PRRSV immunohistochemistry (IHC) staining in brain tissues at 10 days post inoculation (DPI). The inoculation groups are shown at the top. Histopathological changes and PRRSV IHC staining in brain tissues are exemplified in (**A**–**F**) and (**G**–**L**), respectively.

Since brain homogenates from numerous virus-inoculated pigs were PRRSV PCR-positive, we further investigated the microscopic lesions and PRRSV IHC staining in brain tissues, with results summarized in Table 3 and Figure 8. The data suggested that pigs inoculated with PRRSV isolates, such as 1-4-4 L1C.5, 1-4-4 L1A, 1-4-4 L1H, and 1-7-4 L1A, developed encephalitis to different degrees, which was corroborated by positive IHC staining and relatively low PCR C_T_ values. This was found not only in virus-inoculated pigs but also in the contact pigs of the 1-4-4 L1A, 1-4-4 L1H, and 1-7-4 L1A groups. Neurological signs including ataxia, incoordination, posterior paresis, and convulsion were observed in four pigs; these included two from 1-4-4 L1A group (one inoculated pig (#5) showing signs at 12 DPI and one contact pig (#38) showing signs at 15 DPC) and two from 1-4-4 L1H group (one inoculated pig (#33) showing signs at 9 DPI and one contact pig (#37) showing signs at 10 DPC). However, in the 1-4-4 L1C.1 group, regardless of inoculated pigs or contact pigs, no infection in brain tissue was observed by PRRSV IHC staining, although some pigs were PRRSV PCR-positive with relatively high C_T_ values (26.3–30.5). Bacteria culture in brain tissue homogenates revealed no growth of bacteria in most pigs (Table 3).

We further examined the possible correlation between PRRSV IHC scores and PCR C_T_ values in brain tissues. In the 1-4-4 L1C.5 group, two inoculated pigs had a PRRSV IHC score of 1, and one inoculated pig had a PRRSV IHC score of 3, with the corresponding PRRSV PCR C_T_ values of 27.9, 20.2 and 17.5 (Table 3). In the 1-4-4 L1A group, three inoculated pigs had a PRRSV IHC score of 1 with C_T_ values ranging from 24.1 to 33.9, and one contact pig had a PRRSV IHC score of 2 with a C_T_ of 22.3. In the 1-4-4 L1H group, two inoculated pigs had a PRRSV IHC score of 2 with C_T_ values ranging from 21.5 to 22.9, and one contact pig had a PRRSV IHC score of 3 with a C_T_ of 20.3. In the 1-7-4 L1A group, two inoculated pigs had a PRRSV IHC score of 1 with C_T_ values ranging from 20.1 to 24.9, and one contact pig had a PRRSV IHC score of 1 with a C_T_ of 20.8. All of the four pigs with neurological signs (pigs #5, 38, 33, and 37) showed infection in brain by IHC, with the scores ranging from 1 to 3 and PRRSV C_T_ values from 20.3 to 24.1. Although some brain homogenates in the 1-4-4 L1C.1-inoculated and contact pigs were PCR-positive, no brain infection was detected by PRRSV IHC. The overall trend is that brain tissues with lower PCR C_T_ values were more likely to be PRRSV IHC-positive, but there could be some exceptions.

### 3.7. Antibody Responses in Inoculated Pigs

**Figure 9:**
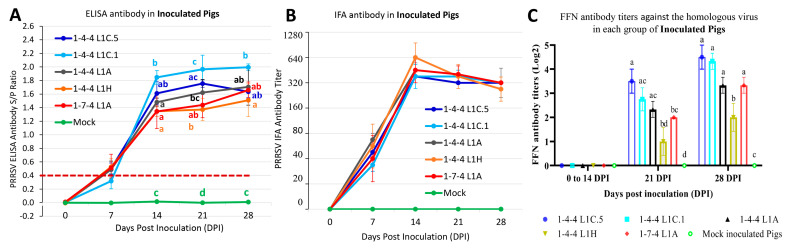
PRRSV antibody responses in serum samples of inoculated pigs over time. (**A**) PRRSV ELISA antibody detected by HerdChek^®^ PRRS X3 ELISA (IDEXX). The average PRRSV ELISA antibody serum to positive (S/P) ratio of each group in serum samples at each time point is shown on the *Y*-axis. (**B**) PRRSV IFA antibody detected in serum samples of inoculated pigs. The average PRRSV IFA antibody titer of each group in serum samples at each time point is shown on the *Y*-axis. (**C**) Homologous PRRSV fluorescent focus neutralization (FFN) antibody titer (log_2_ format) is shown on the *Y*-axis. The statistical analysis was conducted between groups at each time point, with significance denoted by letters. Labels with different letters indicate significant differences; for example, a and b have a significant difference, but a and ab have no significant difference.

PRRSV ELISA antibody results are shown in Figure 9A. All pigs in the six inoculation groups were seronegative at 0 DPI. All pigs in the mock-inoculated group were negative throughout the study. At 7 DPI, three out of four pigs were seropositive in the 1-4-4 L1C.5 and 1-4-4 L1H inoculation groups, two out of four pigs were seropositive in the 1-4-4 L1A and 1-7-4 L1A inoculation groups, and one out of four pig was seropositive in 1-4-4 L1C.1 inoculation group. There was no significant difference in ELISA S/P ratios between the virus-inoculated groups. At 14, 21, and 28 DPI, all pigs in each virus inoculation group were seropositive. At 14 DPI, the 1-4-4 L1C.1 group had significantly higher ELISA S/P ratios than all other virus inoculation groups except the 1-4-4 L1C.5 inoculation group. Similarly, the 1-4-4 L1C.1 inoculation group had substantially higher ELISA S/P ratios than the 1-4-4 L1H inoculation group at 21 and 28 DPI, and had a significantly higher ELISA S/P ratio than the 1-7-4 L1A inoculation group at 21 DPI.

PRRSV IFA antibody responses were determined in serum samples of inoculated pigs using the PRRSV-2 ISU-P isolate as the indicator virus, and the results are summarized in Figure 9B. At 0 DPI, serum samples from all inoculation groups were IFA antibody-negative. At 7 DPI, all pigs in 1-4-4 L1C.1 and 1-4-4 L1A inoculation groups, and three out of four pigs in the 1-4-4 L1C.5, 1-4-4 L1H, and 1-7-4 L1A inoculation groups were IFA antibody-positive. At 14, 21, and 28 DPI, all pigs in all five virus inoculation groups were IFA antibody-positive. Regarding PRRSV IFA antibody titers, no significant difference was found between virus inoculation groups during 7–28 DPI. All pigs in the mock-inoculated group remained IFA antibody-negative over time and were significantly different from the virus-inoculated groups (*p* < 0.05) at 7, 14, 21, and 28 DPI.

PRRSV homologous FFN antibody testing results for the serum samples of inoculated pigs are shown in Figure 9C. From 0 to 14 DPI, the mock-inoculation group and all five virus inoculation groups were FFN antibody-negative. At 21 DPI, low FFN antibody titers in the range of 1 to 3.5 log2 were detected; the l-4-4 L1C.5 inoculation group had significantly higher FFN antibody titer than 1-4-4 L1H and 1-7-4 L1A groups but not significantly different from the 1-4-4 L1C.1 and 1-4-4 L1A inoculation groups. At 28 DPI, slightly higher FFN antibody titers in the 2–4.5 log2 range were detected; all virus inoculation groups, except the 1-4-4 L1H group, had similar FFN antibody titers.

### 3.8. Outcomes in Contact Pigs

**Figure 10:**
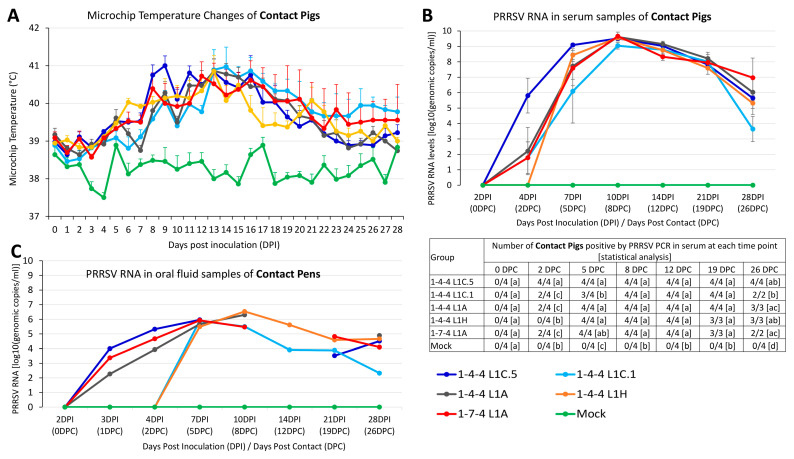
Microchip body-temperature and PCR data in serum and oral fluid samples collected from contact pigs. (**A**) The mean microchip body temperature in degree Celsius is shown on the *Y*-axis for contact pigs over time. (**B**) PRRSV RNA detected in serum samples of contact pigs by quantitative real-time RT-PCR. The number of pigs confirmed positive by PRRSV PCR and statistical analysis are shown in the bottom. The statistical analysis was conducted between groups at each time point, with significance denoted by letters. Labels with different letters indicate significant differences; for example, a and b have a significant difference, but a and ab have no significant difference. (**C**) PRRSV RNA log_10_(genomic copies/mL) in pen-based oral fluids of contact pigs. Oral fluids could not be collected at some time points from some groups due to the inactivity of pigs.

Microchip temperature data from the contact pigs are summarized in Figure 10A. The 1-4-4 L1C.5 contact group had significantly higher average temperatures compared to the 1-4-4 L1C.1 and 1-4-4 L1A contact groups at 8 DPI (6 DPC). Similarly, the 1-4-4 L1C.5 contact group had significantly higher average temperatures than the 1-4-4 L1C.1, 1-4-4 L1H, and 1-7-4 L1A contact groups at 9 DPI (7 DPC) and significantly higher average temperatures than the 1-4-4 L1C.1 and 1-7-4 L1A contact groups at 11 DPI (9 DPC). All the virus-contact groups had significantly higher average temperatures than the mock-contact group at the time points 3, 4, 8–16, 18–23, and 27 DPI.

All contact pigs were PCR-negative on introduction, i.e., 2 DPI or 0 DPC (Figure 10B). All contact pigs (4/4) in the 1-4-4 L1C.5 group become PRRSV PCR-positive at 2 DPC. In contrast, at 2 DPC, only two out of four contact pigs in the 1-4-4 L1C.1, 1-4-4 L1A, and 1-7-4 L1A groups were PRRSV PCR-positive, while no contact pig in the 1-4-4 L1H group was PRRSV PCR-positive. At 2 DPC, the 1-4-4 L1C.5 virus-contact group had significantly higher viremia levels than all other virus-contact groups. At 5 DPC, the 1-4-4 L1C.5 virus-contact group had significantly higher viremia levels than the 1-4-4 L1C.1 contact group but had no significant differences with the other three virus-contact groups. No statistically significant difference was found between virus-contact groups at 8, 12, and 19 DPC. At 26 DPC, 1-7-4 L1A and 1-4-4 L1A contact pigs had significantly higher viremia levels than the 1-4-4 L1C.1 contact group. Based on PRRSV PCR data on the oral fluid samples, the 1-4-4 L1C.5, 1-7-4 L1A, and 1-4-4 L1A contact pens started to become PCR-positive at 1 DPC, whereas the 1-4-4 L1C.1 and 1-4-4 L1H contact pens did not become PCR-positive until 5 DPC (Figure 10C). Similar to the viremia level, the 1-4-4 L1C.5 contact pen had a numerically higher virus load than all other virus-contact pens at 1 DPC and 2 DPC (Figure 10C). Notably, no statistical analysis was conducted on viral loads in oral fluid samples because there was only one oral fluid sample from each contact pen at each time point. During 8–19 DPC, no oral fluid was collected from some virus-contact pens. All pigs in the mock-contact group remained PRRSV PCR-negative throughout the study.

**Figure 11:**
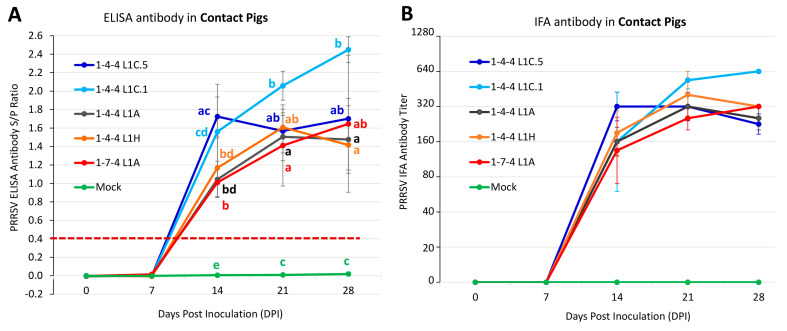
PRRSV antibody responses in contact pigs. (**A**) PRRSV ELISA antibody detected in serum samples of contact pigs. (**B**) PRRSV IFA antibody detected in serum samples of contact pigs. The statistical analysis was conducted between groups at each time point, with significance denoted by letters. Labels with different letters indicate significant differences; for example, a and b have a significant difference, but a and ab have no significant difference.

As shown in Figure 11A, all contact pigs were ELISA antibody-negative at 0 DPI (–2 DPC) and 7 DPI (5 DPC). At 14 DPI (12 DPC), all contact pigs were ELISA antibody-positive in the 1-4-4 L1C.5, 1-4-4 L1A, 1-4-4 L1H, and 1-7-4 L1A groups, and three out of four contact pigs were ELISA antibody-positive in the 1-4-4 L1C.1 group. The 1-4-4 L1C.5 contact group had a significantly higher ELISA S/P ratio than the 1-4-4 L1A, 1-4-4 L1H, and 1-7-4 L1A contact groups, but had no significant difference with the 1-4-4 L1C.1 contact group. At 21 and 28 DPI, all pigs were ELISA antibody-positive in all virus-contact groups. At 21 DPI, the 1-4-4 L1C.1 contact group had significantly higher ELISA S/P ratio than the 1-4-4 L1A and 1-7-4 L1A contact groups. Likewise, the 1-4-4 L1C.1 contact group had a considerably higher ELISA S/P ratio than the 1-4-4 L1A and 1-4-4 L1H contact groups at 28 DPI. All pigs in the mock-contact group were ELISA antibody-negative throughout the study.

As shown in Figure 11B, all contact pigs were IFA antibody-negative at 0 DPI (−2 DPC) and 7 DPI (5 DPC). At 14 DPI (12 DPC), all pigs were IFA antibody-positive in the 1-4-4 L1C.5, 1-4-4 L1A, 1-4-4 L1H, and 1-7-4 L1A contact groups, and three out of four pigs were IFA antibody-positive in the 1-4-4 L1C.1 contact group. At 21 and 28 DPI, all pigs were IFA antibody-positive in all virus-contact groups. No significant difference was found between virus-contact groups regarding the PRRSV IFA antibody titers during 14–28 DPI. All pigs in the mock-contact group remained IFA antibody-negative over time and were significantly different from virus-contact groups (*p* < 0.05) at 14 (12 DPC), 21 (19 DPC), and 28 DPI (26 DPC).

### 3.9. Whole Genome Sequence Comparison of PRRSV-2 Isolates Evaluated in This Study

The whole genome sequences of the PRRSV-2 isolates USA/MN/01775GA/2021 (1-4-4 L1C.5), USA/NE/05828-3/2020 (1-4-4 L1C.1), USA/85099/2018 (1-4-4 L1A), USA/81793-6/2019 (1-4-4 L1H), and USA/IN/65239GA//2014 (1-7-4 L1A) propagated in ZMAC cells were determined and compared to the PRRSV-2 prototype isolate VR-2332.

The nucleotide identities at the whole genome level and the individual nsp and structural protein genes, and the amino acid identities at the protein level between the 1-4-4 L1C.5 isolate MN/01775GA/2021 and other evaluated PRRSV-2 isolates are summarized in Supplemental Table S1. For example, the nucleotide identities ranged from 83.5% to 89.3% at the whole genome level, 76.1% to 88.3% at nsp2, and 86.2% to 92.4% at ORF5.

The nucleotide length of 5′ UTR, each ORF, 3′ UTR, and the amino acid length of the proteins encoded by the respective ORF are summarized in Table 4. Compared to the VR-2332 isolate, the length differences were mainly found in the 5′ UTR, 3′ UTR, and nsp2 regions of other five PRRSV-2 lineage 1 isolates. In addition, some nucleotide point mutations in ORF5a resulted in early translation stop codons and shorter ORF5a proteins on some virus isolates (Table 4). The lengths of nucleotides in nsp1, nsp3–nsp12, ORF2a, ORF2b, ORF3, ORF4, ORF5, ORF6, ORF7, and their corresponding encoded proteins are the same among the VR-2332 isolate and the PRRSV-2 lineage 1 isolates evaluated in this study (Table 4).

**Figure 12:**
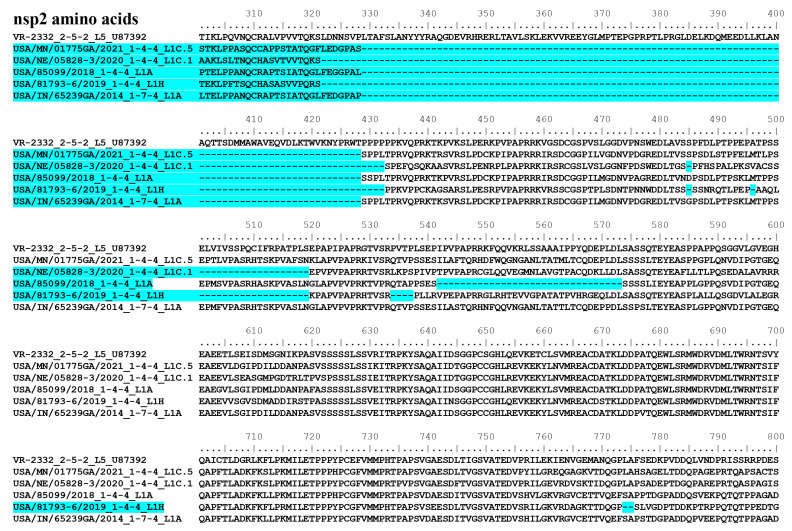
Partial nsp2 protein sequences of five PRRSV-2 isolates included in this study in comparison with the PRRSV-2 prototype isolate VR-2332. The positions evident in the figure represent positions of the nsp2 amino acid sequence in reference to that of VR-2332. The deletion of the amino acids in each PRRSV isolate is highlighted in blue.

As shown in Figure 12, compared to the PRRSV-2 prototype isolate VR-2332, both the 1-4-4 L1C.5 and 1-7-4 L1A isolates had a continuous deletion of 100 amino acids in the nsp2 protein (positions 329–428 according to the location in VR-2332). The 1-4-4 L1C.1 isolate had discontinuous deletions of 111 amino acids, 1 amino acid and 19 amino acids; the 1-4-4 L1A isolate had discontinuous deletions of 100 amino acids and 32 amino acids; and the 1-4-4 L1H isolate had discontinuous deletions of 111 amino acids, 1 amino acid, 1 amino acid, 19 amino acids, 4 amino acids, and 2 amino acids, at different positions of nsp2 when compared to the VR-2332 isolate. The nucleotide insertions or deletions at 5′ UTR and 3′ UTR are shown in Supplemental Figure S2 with unknown functional significance. For example, the 1-4-4 L1C.1 isolate NE/05828-3/2020 had a continuous 10-nucleotide deletion compared to the other five PRRSV-2 isolates (including VR-2332) in the 3′ UTR. Regarding the ORF5a protein, the 1-4-4 L1A isolate 85009/2018 had the same length of 51 amino acids as the VR-2332 isolate, whereas the 1-4-4 L1C.5 isolate MN/01775GA/2021, the 1-4-4 L1C.1 isolate NE/05828-3/2020, 1-4-4 L1H isolate 81793-6/2019, and the -7-4 L1A isolate IN/65239GA/2014 had a length of 46 amino acids, which is shorter than those of VR-2332 and 1-4-4 L1A 85009/2018 isolates.

The number and locations of the predicted N-glycosylation sites in GP2 and GP3 proteins were conserved among the VR-2332 isolate and five PRRSV-2 lineage 1 isolates evaluated in this study (Supplemental Figure S2). In contrast, there were some differences in GP4 and GP5 proteins among these virus isolates. For example, the 1-4-4 L1C.1 isolate NE/05828-3/2020 had one additional predicted N-glycosylation site “NPS” located at GP4 amino acid residues 57–59 when compared to the other five virus isolates, including VR-2332. In the GP5 protein, the N-glycosylation sites at residues 44 and 51 were conserved among all six PRRSV-2 isolates in this study, while the potential N-glycosylation sites at other amino acid residues 30, 32, 33, 34, 35, and 57 varied among the isolates (Supplemental Figure S2).

### 3.10. Growth Characteristics of Virus Isolates in ZMAC Cells

**Figure 13:**
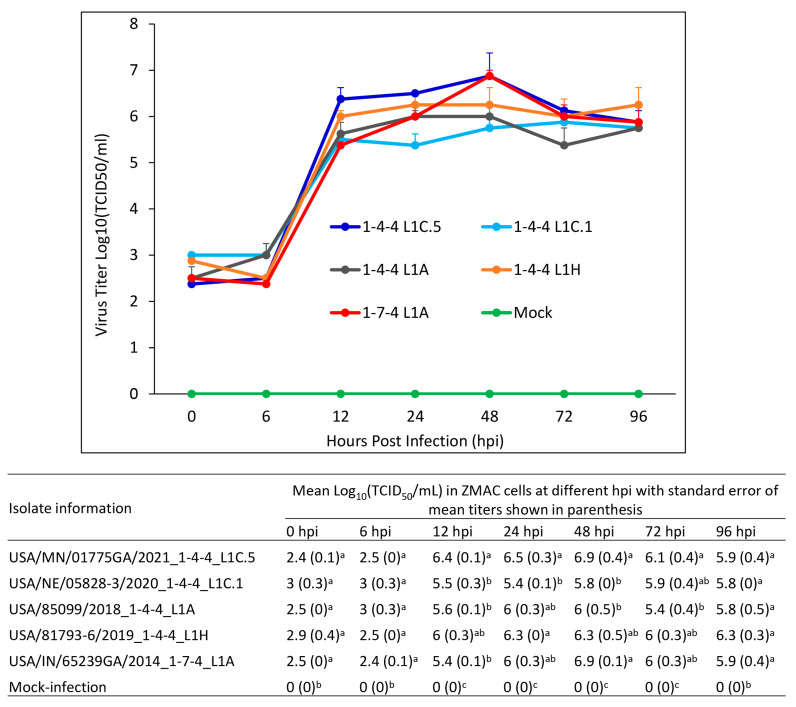
Multistep growth curve analysis of five PRRSV-2 isolates in ZMAC cells. (**Top**) The virus titers Log_10_(TCID_50_/mL) are shown on the *Y*-axis with the standard error of the mean presented. (**Bottom**) The statistical analysis was conducted on the mean Log_10_(TCID_50_/mL) between groups at each hour post infection (hpi). Labels with different letters indicate significant differences; for example, a and b have a significant difference, but a and ab have no significant difference.

The growth characteristics of five PRRSV-2 lineage 1 isolates evaluated in this study were compared in ZMAC cells, and the growth curve is shown in Figure 13. At 0 hpi, the baseline mean titers of the viruses ranged from 2.4 to 3 log_10_ (TCID50/mL) but with no significant titer differences. At 6 hpi, the mean virus titers ranged from 2.4 to 3 log_10_ (TCID50/mL), with no significant differences among the virus isolates. At 12 hpi, the mean virus titers ranged from 5.4 to 6.4 log_10_ (TCID50/mL); the mean titer of 1-4-4 L1C.5 isolate was significantly higher than the 1-4-4 L1C.1, 1-4-4 L1A and 1-7-4 L1A isolates, but no significant difference was observed between the 1-4-4 L1C.5 and 1-4-4 L1H isolates. At 24 hpi, the mean virus titers ranged from 5.4 to 6.5 log_10_ (TCID50/mL); the mean titer in the 1-4-4 L1C.5 isolate was significantly higher than the 1-4-4 L1C.1 isolate but not significantly different from the 1-4-4 L1A, 1-4-4 L1H and 1-7-4 L1A isolates. At 48 hpi, the mean virus titers ranged from 5.8 to 6.9 log_10_ (TCID50/mL); the mean titers in the 1-4-4 L1C.5 and 1-7-4 L1A isolates were significantly higher than the 1-4-4 L1C.1 and 1-4-4 L1A isolates but not significantly different from the 1-4-4 L1H isolate. At 72 hpi, the mean virus titer in the 1-4-4 L1C.5 isolate titer was significantly higher than the 1-4-4 L1A isolate but had no significant difference with the 1-4-4 L1C.1, 1-4-4 L1H, and 1-7-4 L1A isolates. At 96 hpi, the mean virus titers ranged from 5.8 to 6.3 log_10_ (TCID50/mL), with no significant differences between the virus isolates.

## 4. Discussion

PRRSV-2 lineage 1 is currently the most dominant lineage in the USA [5]. Also, PRRSV-2 lineage 1 was found to be susceptible to recombination among PRRSVs in the USA and China [28], which may also contribute to virulence differences of PRRSV-2 isolates. The current study compared the virulence, transmissibility, and antibody responses of five PRRSV-2 lineage 1 isolates in 4-week-old PRRSV-naive weaned pigs. These isolates include the recently emergent 1-4-4 L1C.5 (L1C variant) isolate, a 1-4-4 L1C.1 isolate, a 1-4-4 L1A isolate, a 1-4-4 L1H isolate, and a 1-7-4 L1A isolate. All of these lineage 1 isolates are currently circulating in the swine population in the USA [5].

The results of this study indicate that there are significant differences between 1-4-4 L1C.5 and other PRRSV-2 isolates evaluated in this study. Pigs inoculated with the 1-4-4 L1C.5 isolate became more lethargic, were off-feed faster, and had higher mortality and lower ADG than other virus-inoculated groups. These findings align with field veterinarians’ observations for the L1C.5 (L1C variant) outbreaks [50]. The 1-4-4 L1C.5 virus-inoculated group had a higher percentage of pigs with fever (>40 °C) during 0–10 DPI and had significantly higher average temperatures than other virus-inoculated groups at several time points, such as 8, 9, and 10 DPI. The 1-4-4 L1C.5 virus-inoculated group had a significantly higher viremia level measured by PRRSV PCR than all other virus-inoculated groups at 2 DPI. More severe gross and microscopic lung lesions were observed in pigs inoculated with the 1-4-4 L1C.5 or 1-7-4 L1A isolates and were significantly higher compared to the 1-4-4 L1C.1, 1-4-4 L1A, and 1-4-4 L1H virus-inoculated groups. Collectively, the 1-4-4 L1C.5 isolate appeared to be more virulent than the 1-7-4 L1A, 1-4-4 L1C.1, 1-4-4 L1A, and 1-4-4 L1H isolates in the weaned pig model. The virulence of the 1-7-4 L1A isolate appeared to be between the 1-4-4 L1C.5 isolate and the other three isolates.

Virulence is a comparative or quantitative term that defines the severity or intensity of the disease caused by the pathogen in the infected individuals [51,52]. In the case of PRRSV, the terms “virulent” and “highly virulent” have been used loosely to describe different PRRSV isolates because there are no unequivocal quantitative criteria to define virulence phenotypes of PRRSV isolates. Ruedas-Torres et al. 2021 [53] have proposed some criteria to define PRRSV virulence, but those have not been widely accepted for use. They believe that correlates of PRRSV virulence could include the following: (1) viral genetic determinants of virulence; (2) clinical signs, temperature, and lesions; (3) wider tissue distribution and higher viral load; (4) higher and expanded tissue tropism; and (5) immunological aspects [53]. Generally speaking, “virulent” or “highly virulent” PRRSV isolates may cause increased mortality and morbidity; severe clinical signs, such as elevated fever, severe respiratory scores, anorexia and lethargy, higher viremia and high viral load in different tissues, severe interstitial pneumonia associated with the involvement of secondary bacterial infections; and altered innate and adaptive immune responses [53]. Some parameters seem to correlate with the PRRSV virulence phenotype. For example, high fever, anorexia, and lethargy are generally observed in pigs infected with virulent PRRSV strains [20,53,54,55,56,57,58,59]. Viremia levels have been reported to be higher for infections with virulent PRRSV-1 isolates or PRRSV-2 isolates [53,59,60]; however, the PRRSV-1 isolate SU1-bel induced lower and shorter viremia in comparison with the reference Lelystad isolate, although SU1-bel isolate caused a greater clinical score and gross lung lesions [61]. However, regarding the immunological parameters, there have been controversial reports between cytokine production and the virulence of PRRSV isolates [53]. Nonetheless, how to consistently define the “virulence” criteria for PRRSV isolates remains to be determined.

Another interesting aspect is that this study provides evidence that some PRRSV isolates may replicate better in the central nervous system than other isolates, allowing the virus to induce neurologic clinical signs and brain infection. We found that all of the L1 PRRSV-2 isolates used in this study, except the 1-4-4 L1C.1 isolate, were able to induce brain infection, which was confirmed by lesions of viral encephalitis and PRRSV immunohistochemistry. This is an area that warrants further studies.

In the current study, a higher number of contact pigs in the 1-4-4 L1C.5 group became viremic at 2 days post contact, implying that the L1C.5 isolate may have higher transmissibility than the other PRRSV-2 lineage 1 isolates evaluated in this study. Higher viremia levels and higher viral load in oral fluid samples were observed at the initial DPI in both the virus-inoculated and contact pens of the 1-4-4 L1C.5 group, supporting this conclusion. However, the transmissibility of the 1-4-4 L1C.5 virus needs to be confirmed with a study involving more pigs because our study used only four contact pigs per group.

This study showed that the routinely used IDEXX PRRSV ELISA antibody assay can detect antibodies against the emergent 1-4-4 L1C.5 isolate. For pigs being exported from the USA to some countries, the PRRSV IFA antibody assay is still used. After the emergence of 1-4-4 L1C.5 (L1C variant) in October 2020, we received inquiries whether the routinely conducted PRRSV IFA antibody assay can detect antibodies against the 1-4-4 L1C.5 virus strain. The data in this study demonstrated that IFA antibody assay based on the ISU-P isolate can readily detect antibodies against the 1-4-4 L1C.5 isolate. All five lineage 1 isolates evaluated in this study only induced a low level of homologous neutralizing antibodies during 21–28 DPI.

It is well accepted that the virulence of PRRSV isolates correlates with some genetic determinants of virulence present in the PRRSV genome. However, the genetic determinants of virulence of PRRSV appear to involve multiple genes and have not been fully understood. Whole genome sequence analyses have revealed high genetic variations in the nsp2 region. In a recent study, based on the systematic analysis of insertion and deletion patterns of nsp2 in PRRSV-2 sequences, five large patterns and 25 subdivided groups have been suggested [28]. Compared to the VR-2332 isolate that belongs to the nsp2 pattern 1.0 [28], the 1-4-4 L1C.5, 1-7-4 L1A, and 1-4-4 L1A fall close to the nsp2 pattern 3.0 with the continuous deletion of 100 amino acids. The 1-4-4 L1C.1 isolate used in this study falls close to the nsp2 pattern 2.1, and the 1-4-4 L1H isolate falls close to the nsp2 pattern 2.4.1. The deletion patterns in nsp2 observed in the PRRSV isolates used in our study were different from the HP-PRRSV strain associated with the 2006 epidemic of atypical PRRSV outbreak in China, which had 1 amino acid and 29 amino acid deletions in the nsp2 region and belonged to the nsp2 pattern 4.0 [20,28]. Moreover, the HP-PRRSV strain associated with the 2006 PRRSV outbreak in China belongs to lineage 8 [22], but the PRRSV isolates evaluated in this experimental study belong to lineage 1 (L1A, L1C.5, L1C.1, and L1H). The nsp2 protein is the most variable non-structural protein among different PRRSV strains [62]. Besides the protease activity of the papain-like protease 2 (PLP2) domain at N-terminal [63,64], nsp2 may play a role in PRRSV pathogenesis and modulate host immune responses by its deubiquitinating activities to inhibit ubiquitin-dependent antiviral pathways [65] and regulate the ubiquitin-dependent innate immunity of type I IFN activation [66]. However, the exact role of nsp2 in contributing to the virulence differences of various PRRSV isolates remains to be determined. In addition to nsp2, considerable variations were observed in other genomic regions and proteins among the PRRSV-2 isolates evaluated in this study (1-4-4 L1C.5, 1-4-4 L1C.1, 1-4-4 L1A, 1-4-4 L1H, and 1-7-4 L1A); the genetic determinants responsible for virulence differences of these PRRSV-2 isolates are still unclear. PRRSV strains with distinct glycosylation patterns may alter the immunogenicity of the virus and induce different neutralizing antibody production [67]. A recent study revealed the different N-glycosylation patterns associated with the emergence of new genetic variants of PRRSV-2 in the USA [68]. The PRRSV-2 isolates evaluated in our current study also have different N-glycosylation patterns, but their correlation with virus phenotypic functions remains to be determined.

When five PRRSV-2 lineage 1 isolates evaluated in this study were compared for their growth in ZMAC cells, the L1C.5 isolate replicated to higher titers at 12, 24 and 48 hpi compared to some other isolates. However, more studies are needed to determine whether growth characteristics of PRRSV isolates in ZMAC cells can reliably predict the virulence of PRRSV isolates.

## 5. Conclusions

This study provides experimental data in weaned pigs regarding the clinical impact, virulence, transmissibility, and antibody detection of the newly emergent L1C.5 virus (1-4-4 L1C variant), along with comparisons with other PRRSV-2 lineage 1 isolates. The findings confirm that L1C.5 virus is highly virulent in weaned pigs. The higher number of contact pigs becoming viremic at 2 days post contact implies that the L1C.5 virus may have higher transmissibility than other PRRSV isolates, although it needs to be confirmed in a study involving more pigs. Brain infection by PRRSV is also an area needing further investigation. The whole genome sequence comparisons of five PRRSV-2 lineage 1 isolates revealed variations in numerous genomic regions at both the nucleotide and amino acid levels, but the genetic determinants for virulence differences of these virus isolates remain to be elucidated. The L1C.5 isolate replicated to higher titers in ZMAC cells at some time points compared to other PRRSV-2 lineage 1 isolates evaluated in this study. However, more studies are needed to determine whether growth characteristics of PRRSV isolates in ZMAC cells can reliably predict the virulence of PRRSV isolates.

## Figures and Tables

**Table 1:** Information of PRRSV isolates used in this study.

Isolate ID	Passage No.	RFLP and Lineage	PRRSV PCR C_T_	TCID_50_/mL in ZMAC Cells	TCID_50_/mL in MARC-145 Cells
USA/MN/01775GA/2021	P4	1-4-4 L1C.5 (L1C variant)	16.5	10^6.75^	10^4.25^
USA/NE/05828-3/2020	P4	1-4-4 L1C.1	17.1	10^6^	10^3.75^
USA/85099/2018	P4	1-4-4 L1A	15.1	10^6.25^	10^3.75^
USA/81793-6/2019	P4	1-4-4 L1H	21.3	10^6.5^	10^3.5^
USA/IN/65239GA/2014	P4	1-7-4 L1A	15.3	10^6.25^	10^4.75^

Abbreviation: TCID_50_/mL: 50% tissue culture infectious dose per mL.

**Table 2:** Proportion of mortality observed in pigs in different virus inoculated groups.

Inoculation Group	Mortality	Occurrence Day
1-4-4 L1C.5 (L1C variant)	6/8	1 (8 DPI); 1 (9 DPI); 2 (10 DPI); 2 (14 DPI)
1-4-4 L1C.1	1/8	1 (20 DPI)
1-4-4 L1A	1/8	1 (12 DPI)
1-4-4 L1H	2/8	2 (9 DPI)
1-7-4 L1A	2/8	1 (9 DPI); 1 (15 DPI)
Mock-inoculation	0/8	

Note: The pigs that naturally died or were euthanized due to the severe body conditions were counted as mortality.

**Table 3:** Summary of pig brain infection after experimental inoculation with different PRRSV isolates.

Group	Pig ID	Euthanasia Date	Brain				Ataxia or Could Not Stand Up
			PRRSV PCR C_T_ in Brain	PRRSV IHC Score in Brain	Average Microscopic Lesion Score *	Bacterial Culture	
1-4-4 L1C.5, Inoculated Pigs	6	8 DPI	×	×	×	×	
	11	10 DPI	27.9	1	1	No growth	
	16	9 DPI	17.5	3	1.75	Low Strep equisimilis	
	63	10 DPI	22.3	0	0.75	No growth	
	20	14 DPI	20.2	1	1	No growth	
	69	14 DPI	34.0	0	0	No growth	
	64	28 DPI	≥40	0	0.5	No growth	
	71	28 DPI	≥40	0	0.5	No growth	
1-4-4 L1C.5, Contact Pigs	22	28 DPI	36.2	0	0	No growth	
	44	28 DPI	34.3	0	0	No growth	
	46	28 DPI	≥40	0	0	No growth	
	55	28 DPI	≥40	0	0.25	No growth	
1-4-4 L1C.1, Inoculated Pigs	7	10 DPI	≥40	0	0.25	No growth	
	23	10 DPI	≥40	0	0.75	No growth	
	70	10 DPI	28.0	0	0.5	No growth	
	72	10 DPI	28.0	0	0.25	No growth	
	12	20 DPI	26.3	0	0.5	No growth	
	19	28 DPI	≥40	0	0.25	No growth	
	66	28 DPI	30.5	0	0	No growth	
	68	28 DPI	≥40	0	0.25	No growth	
1-4-4 L1C.1, Contact Pigs	30	28 DPI	30.3	0	0.5	No growth	
	35	28 DPI	30.3	0	0.25	No growth	
	43	28 DPI	≥40	0	0	No growth	
	47	28 DPI	≥40	0	0.25	No growth	
1-4-4 L1A, Inoculated Pigs	1	10 DPI	≥40	0	0.5	Low Strep suis	
	13	10 DPI	33.3	0	0	No growth	
	53	10 DPI	25.0	1	1	No growth	
	65	10 DPI	33.9	1	0.5	No growth	
	5	12 DPI	24.1	1	0.75	No growth	Yes
	8	28 DPI	30.4	0	0	No growth	
	58	28 DPI	36.7	0	0	No growth	
	59	28 DPI	≥40	0	0	No growth	
1-4-4 L1A, Contact Pigs	38	17 DPI	22.3	2	1.5	No growth	Yes
	21	28 DPI	≥40	0	0	No growth	
	39	28 DPI	≥40	0	0	No growth	
	40	28 DPI	29.8	0	0.25	No growth	
1-4-4 L1H, Inoculated Pigs	17	10 DPI	22.9	2	1.25	No growth	
	33	9 DPI	21.5	2	3	No growth	Yes
	45	10 DPI	34.2	0	0.25	No growth	
	62	9 DPI	×	×	×	×	
	10	28 DPI	≥40	0	0	No growth	
	18	28 DPI	37.9	0	0.25	No growth	
	49	28 DPI	35.3	0	0.5	No growth	
	57	28 DPI	≥40	0	0.5	No growth	
1-4-4 L1H, Contact Pigs	37	12 DPI	20.3	3	2.25	No growth	Yes
	34	28 DPI	≥40	0	0.25	No growth	
	41	28 DPI	34.8	0	0.75	No growth	
	42	28 DPI	31.9	0	0.25	No growth	
1-7-4 L1A, Inoculated Pigs	26	9 DPI	×	×	×	×	
	27	10 DPI	24.9	1	1.5	Few Glaesserella parasuis	
	60	10 DPI	35.1	0	0.5	No growth	
	61	10 DPI	28.8	0	0	No growth	
	4	15 DPI	20.1	1	1.5	No growth	
	2	28 DPI	≥40	0	0	No growth	
	52	28 DPI	≥40	0	0.25	No growth	
	67	28 DPI	≥40	0	0.25	No growth	
1-7-4 L1A, Contact Pigs	36	12 DPI	20.8	1	1.25	No growth	
	50	15 DPI	30.1	0	0.25	No growth	
	48	28 DPI	≥40	0	0.25	No growth	
	51	28 DPI	25.0	0	0.25	No growth	
Mock-inoculated Pigs	9	10 DPI	≥40	0	0	No growth	
	15	10 DPI	≥40	0	0	No growth	
	31	10 DPI	≥40	0	0	No growth	
	56	10 DPI	≥40	0	0	No growth	
	3	28 DPI	≥40	0	0	Low Strep suis	
	14	28 DPI	≥40	0	0	No growth	
	32	28 DPI	≥40	0	0	No growth	
	54	28 DPI	≥40	0	0	No growth	
Mock-contact Pigs	24	28 DPI	≥40	0	0	No growth	
	25	28 DPI	≥40	0	0	No growth	
	28	28 DPI	≥40	0	0	No growth	
	29	28 DPI	≥40	0	0	No growth	

* Meningitis, gliosis, vasculitis/perivasculitis, and neuronal necrosis were each scored in the range of 0–3, and the average score of the four categories of lesions was calculated. Pigs with PRRSV IHC-positive staining in brain are highlighted in green. Pigs with relatively low PCR C_T_ values in brain but negative IHC staining in brain are highlighted in orange. For those pigs marked with ×, brain samples were not collected.

**Table 4:** Summary of genomic sequence comparisons of the five PRRSV isolates evaluated in this study together with VR-2332 isolate.

ORF	Protein	Nucleotide Length (Protein Length)					
		2-5-2 L5A (VR-2332)	1-4-4 L1C.5 (USA/MN/01775 GA/2021)	1-4-4 L1C.1 (USA/NE/05828-3/2020)	1-4-4 L1A (USA/85099/2018)	1-4-4 L1H (USA/81793-6/2019	1-7-4 L1A (USA/IN/65239 GA/2014
5′ UTR	N.A.	189 nt	187 nt	190 nt	188 nt	190 nt	188 nt
ORF1a	pp1a	7512 nt (2503 aa)	7212 nt (2403 aa)	7119 nt (2372 aa)	7116 nt (2371 aa)	7098 nt (2365 aa)	7212 nt (2403 aa)
ORF1b	N.A.	all 4377 nt					
ORF1a/b	pp1ab	11,883 nt (3960 aa)	11,583 nt (3860 aa)	11,490 nt (3829 aa)	11,487 nt (3828 aa)	11,469 nt (3822 aa)	11,583 nt (3860 aa)
	nsp1	all 1149 nt (383 aa)					
	nsp2	3588 nt (1196 aa)	3288 nt (1096 aa)	3195 nt (1065 aa)	3192 nt (1064 aa)	3174 nt (1058 aa)	3288 nt (1096 aa)
	nsp3	all 690 nt (230 aa)					
	nsp4	all 612 nt (204 aa)					
	nsp5	all 510 nt (170 aa)					
	nsp6	all 48 nt (16 aa)					
	nsp7	all 777 nt (259 aa)					
	nsp8	all 135 nt (45 aa)					
	nsp9	all 2055 nt (685 aa)					
	nsp10	all 1323 nt (441 aa)					
	nsp11	all 669 nt (223 aa)					
	nsp12	all 459 nt (153 aa)					
ORF2a	GP2	all 771 nt (256 aa)					
ORF2b	E	all 222 nt (73 aa)					
ORF3	GP3	all 765 nt (254 aa)					
ORF4	GP4	all 537 nt (178 aa)					
ORF5a	ORF5a	156 nt (51 aa)	141 nt (46 aa)	141 nt (46 aa)	156 nt (51 aa)	141 nt (46 aa)	141 nt (46 aa)
ORF5	GP5	all 603 nt (200 aa)					
ORF6	M	all 525 nt (174 aa)					
ORF7	N	all 372 nt (123 aa)					
3′ UTR	N.A.	151 nt	151 nt	141 nt	151 nt	151 nt	151 nt

N.A.—Not applicable. nt—nucleotides. aa—amino acid.

## Data Availability

The raw data are available and can be provided upon request.

## Supplementary Materials

The following supporting information can be downloaded at: https://www.mdpi.com/article/10.3390/v15112233/s1, Figure S1: Microscopic lesions and PRRSV immunohistochemistry (IHC) scores in lung tissues of inoculated pigs at 28 DPI; Figure S2: Comparison of 5′ UTR and 3′ UTR nucleotides and GP2, GP3, GP4, GP5, and ORF5a proteins of six PRRSV-2 isolates; Table S1: Nucleotide and amino acid identities between the L1C.5 isolate MN/01775GA/2021 and other PRRSV isolates.
