# Supplementary material for: In Vivo and In Vitro Characterization of the Recently Emergent PRRSV 1-4-4 L1C Variant (L1C.5) in Comparison with Other PRRSV-2 Lineage 1 Isolates
Source: Viruses. 2023 Nov 9;15(11):2233. doi: 10.3390/v15112233 (PMC10674456; doi:10.3390/v15112233)
Supplement: Supplementary file 1 [file viruses-15-02233-s001.zip › viruses-2673374-supplementary.pdf]

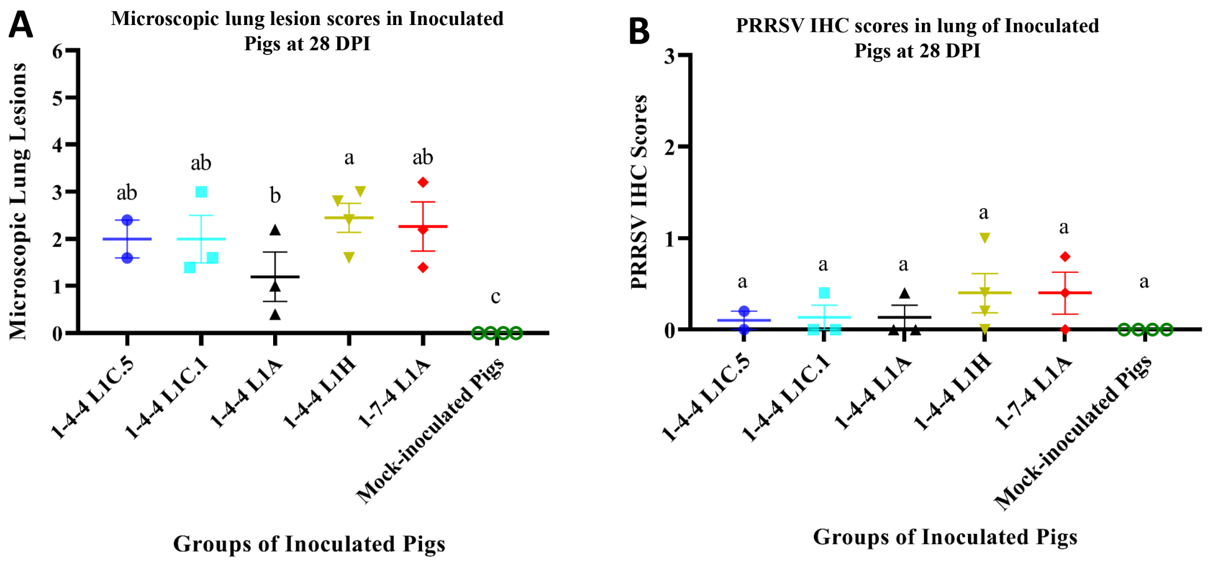

Supplemental Figure S1. Microscopic lesions, and PRRSV immunohistochemistry (IHC) scores in lung tissues of inoculated pigs at 28 DPI. (A) Microscopic lung lesion scores (in the range of 0–6) in inoculated pigs at 28 DPI. (B) PRRSV IHC staining scores (in the range of 0–3) in lung tissues of inoculated pigs at 28 DPI. A cluster graph was used to present the data with standard error of the mean. The statistical analysis was conducted between groups, with significance denoted by letters on the individual plot. Labels with different letters indicate significant differences; for example, a and b have a significant difference, but a and ab have no significant difference.

5’ UTR nucleotides

```
                                             60        70        80        90       100       110       120       130       140       150
                                         ....|....|....|....|....|....|....|....|....|....|....|....|....|....|....|....|....|....|
VR-2332_2-5-2_L5_U87392                  TGTGACCATTGGCACAGCCCAAAACTTGCTGCACAGAAACACCCTTCTGTGATAGCCTCCTTCAGGGGAGCTTAGGGTTT-GTCCCTAGCACCTTGCTTC
USA/MN/01775GA/2021_1-4-4_L1C.5          TGTGACCACTGGCACAGCCCAAA-CTTGCTGCACAGAAACACCCTTCTGTGACAGCCTCCTTCAAGG-AGTTTAGGGGTT-GTCCCTAGCACCTTGTTTC
USA/NE/05828-3/2020_1-4-4_L1C.1          TGTGACCACTGGCACGGCCCAAAACTTGCTGCACGGGAACACCCTCCTGTGACGGCCTCCTCCAGGGAAGTTTAGGGGTCTGTCTCTAACGCCTTGGTTC
USA/85099/2018_1-4-4_L1A                 TGTGACTACTGGCACAGCCCAAAGCTTGCTGCACAGAAACACCCTTCTGTGACGGCCTCCTTCAGGG-AATTTAGGGGTT-GTCCCTAGCACCCTGCTTC
USA/81793-6/2019_1-4-4_L1H               CGTGACCATTGGCACGGCCCAAAACTTGCTGCACGGGAACACCCTCCTGTGACGGCCTCCTTCAGGGGAGTTTAGGGGTTTGTCCCTAACACCTTGTTTC
USA/IN/65239GA/2014_1-7-4_L1A            TGTGACCACTGGCACAGCCCAAAGCTTGCTGCACAGAAACACCCTTCTGTGACGGCCTCCTTCGGGG-AGTTTAGGGGTT-GTCCCTAGCACCCTGCTTC
```

3’ UTR nucleotides

```
                                             10        20        30        40        50        60        70        80        90       100
                                         ....|....|....|....|....|....|....|....|....|....|....|....|....|....|....|....|....|....|....|....|
VR-2332_2-5-2_L5_U87392                  TGGGCTGGCATTCTTGAGGCATCTCAGTGTTTGAATTGGAAGAATGTGTGGTGAATGGCACTGATTGACATTGTGCCTCTAAGTCACCTATTCAATTAGG
USA/MN/01775GA/2021_1-4-4_L1C.5          TGAGCTGGCATTCTTGATACATCCCAGTGTCTGAATTAGAAGAATGAGTGGTGAATGGCACTGATTGACATTGTGCCTCTAAGTCACCTATTCAATTAGG
USA/NE/05828-3/2020_1-4-4_L1C.1          CAAG----------TGGGACATCCCAGTGTTTGAATTGGAAGAATGAGTGGTGAATGGCACTGATTGATATTGTGCCTCTAAGTCACCTATTCAATTAGG
USA/85099/2018_1-4-4_L1A                 TGAGCTGACATTCTTGAGACATCCCAGTGCCTGAATTGGAAGAATGAGTGGTGAATGGCACTGATTGACATTGTGCCTCTAAGTCACCTATTCAATTAGG
USA/81793-6/2019_1-4-4_L1H               TGAGCTGGCATTCTTGAGACATCCCAGTGTTTGAATTGGAAGAATGTGTGGTGAATGGCACTGATTGACATTGTGCCTCTAAGTCACCTATTCAATTAGG
USA/IN/65239GA/2014_1-7-4_L1A            TGAGCTGGCATTCTTGAGACATCCCAGTGCCTGAATTGGAAGAATGAGTGGTGAATGGCACTGATTGACATTGTGCCTCTAAGTCACCTATTCAATTAGG
```

GP2 amino acids

```
                                            110       120       130       140       150       160       170       180       190       200
                                         ....|....|....|....|....|....|....|....|....|....|....|....|....|....|....|....|....|....|....|....|
VR-2332_2-5-2_L5_U87392                  HKVSTLIDEMVSRRMYRIMEKAGQAAWKQVVSEATLSRISSLDVVAHFQHLAAIEAETCKYLASRLPMLHNLRMTGSNVTIVYNSTLNQVFAIFPTPGSR
USA/MN/01775GA/2021_1-4-4_L1C.5          HKVSTLIDEMVSRRMYRTMENAGQAAWKQVVSEATLSRISSLDVVAHFQHLAAIEAETCKYLASRLPMLHNLRLTGSNVTIVYNSTLDQISAIFPTPDSR
USA/NE/05828-3/2020_1-4-4_L1C.1          HKVSTLIDEMVSRRMYRTMENAGQAAWRQVVSEATLSRISSLDVVAHFQHLAAIEAETCKYLASRLPMLHNLRITGSNVTIKYNSTSGRVFAIFPTQHSR
USA/85099/2018_1-4-4_L1A                 HKVSTLIDEMVSRRMYRTMEDAGQAAWKQVVSEATLSRISSLDVVAHFQHLAAIEAETCKYLASRLPMLHNLRMTGSNVTIVYNSTSNRVSAIFPTPGSR
USA/81793-6/2019_1-4-4_L1H               HKVSTLIDEMVSRRIYRSMENAGQAAWKQVVSEATLSRISSLDVVAHFQHLAAIEAETCKYLASRLPMLHNLRMTGSNVTIKYNTTLDRVSAIFPTPDSR
USA/IN/65239GA/2014_1-7-4_L1A            HKVSTLIDEMVSRRMYRTMEQAGQAAWKQVVTEATLSRISSLDVVAHFQHLAAIEAETCKYLASRLPMLHNLRLTGSNVTIVYNSSLDRVFAVFPTSSSR
```

GP3 amino acids

```
                                             30        40        50         130       140       150       160       170       180       190       200
                                         ....|....|....|....|....|....|     |....|....|....|....|....|....|....|....|....|....|....|....|....|....|....|
VR-2332_2-5-2_L5_U87392                  AGSNTTYCFWFPLVRGNFSFELTVNYTVCP     GNVSRVYVDIKHQLICAEHDGQNTTLPRHDNISAVFQTYYQHQVDGGNWFHLEWLRPFFSSWLVLNVSWFL
USA/MN/01775GA/2021_1-4-4_L1C.5          ADSNATFCFWFPLVRGNFSFELFVNYTVCQ     GNVSGVYVDKLHQLICAVHDGQNATLSRYDNITAVYQTYYQHQVDGGNWFHLEWLRPFFSSWLVLNVSWFL
USA/NE/05828-3/2020_1-4-4_L1C.1          TSANSSYCFWFPLVRGNFSFELMVNYTVCP     GNVSEVYVDIQGQLICAVHDGQNATLPSHDNITAVYQTYYQHQVDGGNWFHLEWLRPFFSSWLVLNVSWFL
USA/85099/2018_1-4-4_L1A                 ANSNATFCFWFPLVRGNFSFELLVNYTVCQ     GNVSTVYVDKPRQLICADHDGQNATLSRYDNITAVYQTYYQHQVDGGNWFHLEWLRPFFSSWLVLNVSWFL
USA/81793-6/2019_1-4-4_L1H               TASNNTYCFWFPLVRGNFSFELTINYTICP     GNVSEVYVDRKHQFICAVHDGLNSTLPRHDNISAVFQTYYQHQVDGGNWFHLEWLRPFFSSWLVLNVSWFL
USA/IN/65239GA/2014_1-7-4_L1A            AASNNTYCFWFPLARGNFSFELTVNYTVCP     GNVSEVYVDIKHQFICAVHDGQNTSLPRHDNITAVYQTYYQHQVDGGNWFHLEWLRPFFSSWLVLNVSWFL
```

GP4 amino acids

```
                                             40        50        60        70        80        90       100       110       120       130
                                         ....|....|....|....|....|....|....|....|....|....|....|....|....|....|....|....|....|....|....|....|
VR-2332_2-5-2_L5_U87392                  LADIKTNTTAAASFAVLQDISCLRHRDSASEAIRKIPQCRTAIGTPVYVTITANVTDENYLHSSDLLMLSSCLFYASEMSEKGFKVVFGNVSGIVAVCVNFTSYV
USA/MN/01775GA/2021_1-4-4_L1C.5          LSDIKTNTTSAASSVVLQDISCLRHNYPSSAAFRKTPQCRTAIGTPVYITITANVTDENYLHSSDLLMLSSCLFYASEMSEKGFRVIFGNVSGIVAVCVNFTDYV
USA/NE/05828-3/2020_1-4-4_L1C.1          LSDIKINTTSAASSVVLQDISCLRHGNPSPAAFRKVPQCRTAIGTPVYITVTANVTDENYLHSSDLLMLSSCLFYASEMSEKGFKVIFGNVSGIVAVCVNFTSYV
USA/85099/2018_1-4-4_L1A                 LSDIKTNTTSAPSSVVLQDISCLRHNYPSSAAFRKIPQCRTAIGTPVYITITANVTDENYLHSSDLLMLSSCLFYASEMSEKGFKVIFGNVSGIVAVCVNFTSYV
USA/81793-6/2019_1-4-4_L1H               LSDIKTNTTAAAGFAVLQDISCLRHGHSSSEAFRKSRQCRTAIGTPAYITITANVTDENYLYSSDLLMLSSCLFYASEMSEKGFKVIFGNVSGIVAVCVNFTSYV
USA/IN/65239GA/2014_1-7-4_L1A            LSDIKTNTTAAAGFAVLQDISCLRHSDSSSEATRKSRQCRTAIGTPVYITVTANVTDENYLHSSDLLMLSSCLFYASEMSEKGFKVVFGNVSGIVAVCVNFTSYV
```

GP5 amino acids

```
                                             10        20        30        40        50        60        70        80        90       100
                                         ....|....|....|....|....|....|....|....|....|....|....|....|....|....|....|....|....|....|....|....|
VR-2332_2-5-2_L5_U87392                  MLEKCLTAGCCSRLLSLWCIVPFCFAVLANASNDSSSHLQLIYNLTLCELNGTDWLANKFDWAVESFVIFPVLTHIVSYGALTTSHFLDTVALVTVSTAG
USA/MN/01775GA/2021_1-4-4_L1C.5          MLGKCLTAGCCSQLLFLWCIVPFCFVALVNANNSSSSHLQLIYNLTICELNGTDWLNERFDWAVETFVIFPVLTHIVSYGALTTSHFLDTVGLATVSTAG
USA/NE/05828-3/2020_1-4-4_L1C.1          MLGKCLTAGYCSRLPFLWCIVPFCLAALVNASNGNSSHLQLIYNLTICELNGTDWLDNHFNWAVETFVIFPVLTHIVSYGALTTSHFLDTIGLITVSTAG
USA/85099/2018_1-4-4_L1A                 MSGKCLIAGCCSQFLFLWCIVPFCFVALVNANNGSSSHLQLIYNLTICELNGTDWLNKTFDWAVETFVIFPVLTHIVSYGALTTSHFLDTVGLITVSAAG
USA/81793-6/2019_1-4-4_L1H               MLVKCLTAGYCSQLLFLWCIVPFCFAALVNANSNSSSHLQLIYNLTICELNGTDWLNNKFDWAVETFVIFPVLTHIVSYGALTTSHFLDTVGLITVSTAG
USA/IN/65239GA/2014_1-7-4_L1A            MLGKCLTAGYCSQLPFLWCIVPFCFAALVNASNNSGSHLQLIYNLTICELNGTDWLDRSFDWAVETFVIFPVLTHIVSYGALTTSHFLDTVGLITVSAAG
```

ORF5a amino acids

```
                                             10        20        30        40        50
                                         ....|....|....|....|....|....|....|....|....|....|..
VR-2332_2-5-2_L5_U87392                  MFKYVGEMLDRGLLLAIAFFVVYRAVLFCCARQRQQRQQLPSTADLQLDAM*
USA/MN/01775GA/2021_1-4-4_L1C.5          MFRYVGEMLDRGLLLAIAFFVVYRAVLFCCARQRQQQQQLPFTVDL*
USA/NE/05828-3/2020_1-4-4_L1C.1          MFRYVGEMLDRGLLLAIAFFVVYRAILSCCARQRQQRQQLPFTVDL*
USA/85099/2018_1-4-4_L1A                 MFRYVGEMLDRGLLLAISFFVVYRAVLFCCARQRQQRQQLPFTIDLQPDNM*
USA/81793-6/2019_1-4-4_L1H               MFKHVGEMLDRGLLLAIAFFVVYRAVLFCCARQRQQQQQLPLTIDL*
USA/IN/65239GA/2014_1-7-4_L1A            MFRYVGEMLDRGLLLAIAFFVVYRAILFCCARQRQQQQRLPFTVDL*
```

Supplemental Figure S2. Comparison of 5′ UTR and 3′ UTR nucleotides and GP2, GP3, GP4, GP5, and GP5a proteins of six PRRSV-2 isolates. Partial nucleotide sequences of 5′ UTR and 3′ UTR harboring insertions or deletions are shown. The GP5a proteins with a truncation at the C-terminal of some PRRSV isolates are shown. The predicted N-glycosylation sites on GP2, GP3, GP4 and GP5 proteins are depicted. The first letter “N” and the remaining two letters of each predicted N-glycosylation site are shown by red color font and blue color font, respectively.

Supplemental Table S1. Nucleotide and amino acid identities between the 1-4-4 L1C.5 isolate MN/01775GA/2021 and other PRRSV isolates

ORF	Protein	Nucleotide identity % (amino acid identity %) compared to 1-4-4 L1C.5 isolate USA/MN/01775GA/2021				
		2-5-2 L5A (VR-2332)	1-4-4 L1C.1 (USA/ NE/05828-3/2020)	1-4-4 L1A (USA/ 85099/2018)	1-4-4 L1H (USA/ 81793-6/2019	1-7-4 L1A (USA/ IN/65239GA/2014
Whole genome	N.A.	85.4% (N.A.)	86.4% (N.A.)	89.3% (N.A.)	83.5% (N.A.)	89.1% (N.A.)
5' UTR	N.A.	93.6% (N.A.)	89.8% (N.A.)	95.2% (N.A.)	90.9% (N.A.)	95.7% (N.A.)
ORF1a	pp1a	83.8% (83.1%)	81.5% (82.2%)	86.8% (88.6%)	79.7% (81.0%)	88.1% (89.9%)
ORF1b	N.A.	86.4% (N.A.)	90.9% (N.A.)	89.7% (N.A.)	87.5% (N.A.)	90.9% (N.A.)
ORF1a/b	pp1ab	N.A. (87.8%)	N.A. (88.1%)	N.A. (91.7%)	N.A. (86.7%)	N.A. (93.0%)
	nsp1	83.3% (85.6%)	83.4% (84.8%)	88.9% (89.8%)	83.9% (85.1%)	91.0% (91.1%)
	nsp2	79.7% (73.6%)	78.6% (74.7%)	86.7% (85.3%)	76.1% (72.4%)	88.3% (87.2%)
	nsp3	86.8% (94.8%)	81.6% (90.4%)	86.9% (93.5%)	83.5% (92.6%)	87.5% (93.9%)
	nsp4	92.0% (94.6%)	81.4% (90.7%)	82.5% (91.2%)	80.4% (91.2%)	83.0% (91.7%)
	nsp5	98.9% (95.3%)	81.4% (84.7%)	82.5% (87.6%)	80.0% (85.9%)	84.3% (89.4%)
	nsp6	93.7% (100%)	87.5% (100%)	91.6% (100%)	85.4% (100%)	89.6% (93.7%)
	nsp7	82.2% (88.0%)	87.6% (89.9%)	88.1% (91.9%)	81.3% (85.3%)	88.7% (92.6%)
	nsp8	89.6% (91.1%)	94.1% (93.3%)	94.8% (97.8%)	91.1% (91.1%)	94.8% (97.8%)
	nsp9	88.0% (96.3%)	90.1% (96.9%)	89.3% (96.8%)	87.2% (96.0%)	90.4% (97.8%)
	nsp10	85.6% (95.2%)	90.2% (97.7%)	89.1% (97.7%)	90.8% (97.0%)	90.2% (98.2%)
	nsp11	85.3% (94.1%)	93.3% (96.8%)	89.8% (94.6%)	84.3% (93.3%)	92.4% (96.8%)
	nsp12	84.1% (91.5%)	94.3% (98.0%)	94.5% (96.7%)	84.5% (92.8%)	93.9% (98.7%)
ORF2a	GP2	87.5% (85.9%)	83.4% (82.8%)	94.9% (94.9%)	83.2% (81.6%)	84.2% (80.1%)
ORF2b	E	89.2% (87.7%)	88.3% (91.8%)	97.3% (97.2%)	86.0% (89.0%)	86.9% (86.3%)
ORF3	GP3	83.5% (81.1%)	85.5% (82.7%)	94.2% (91.7%)	81.7% (79.1%)	81.8% (79.1%)
ORF4	GP4	86.0% (85.9%)	93.6% (92.7%)	95.9% (94.4%)	88.8% (88.2%)	88.1% (88.2%)
ORF5a	GP5a	90.1% (91.3%)	91.5% (93.5%)	95.0% (93.5%)	92.2% (91.3%)	92.2% (95.6%)
ORF5	GP5	86.4% (85.0%)	92.4% (91.0%)	88.4% (92.5%)	86.2% (93.0%)	87.9% (90.0%)
ORF6	M	89.3% (93.1%)	96.2% (97.1%)	93.9% (94.2%)	88.6% (94.8%)	94.1% (94.8%)
ORF7	N	89.2% (91.9%)	94.3% (95.1)	95.4% (96.7%)	85.7% (87.0%)	96.5% (97.5%)
3' UTR	N.A.	93.6% (N.A.)	94.3% (N.A.)	96.4% (N.A.)	96.4% (N.A.)	97.1% (N.A.)

N.A. – Not applicable
